# EGF-Coupled Gold Nanoparticles Increase the Expression of CNPase and the Myelin-Associated Proteins MAG, MOG, and MBP in the Septal Nucleus Demyelinated by Cuprizone

**DOI:** 10.3390/life12030333

**Published:** 2022-02-23

**Authors:** Eduardo Lira-Diaz, Jesus Monroy-Rodriguez, Maria G. Gonzalez-Pedroza, Raul A. Morales-Luckie, Luis Castro-Sánchez, Oscar Gonzalez-Perez

**Affiliations:** 1 Laboratory of Neuroscience, School of Psychology, University of Colima, Colima 28040, Mexico; elira@ucol.mx; 2 Physiological Science PhD Program, School of Medicine, University of Colima, Colima 28040, Mexico; 3 University Center for Biomedical Research, University of Colima, Colima 28045, Mexico; jmonroy@ucol.mx (J.M.-R.); luis_castro@ucol.mx (L.C.-S.); 4 Department of Biotechnology, School of Sciences, Autonomous University of the State of Mexico, Toluca 50200, Mexico; mggonzalezp@uaemex.mx; 5 Department of Nanomaterials, Joint Center for Research in Sustainable Chemistry UAEMex—UNAM (CCIQS), Autonomous University of the State of Mexico (UAEMex), Toluca 50200, Mexico; rmoralesl@uaemex.mx; 6 CONACyT-University of Colima, Colima 28045, Mexico

**Keywords:** epidermal growth factor, remyelination, gold nanoparticles, CNPase, MAG, MOG, MBP

## Abstract

Current pharmacological therapies against demyelinating diseases are not quite satisfactory to promote remyelination. Epidermal growth factor (EGF) can expand the population of oligodendrocyte precursor cells (OPCs) that may help with the remyelination process, but its delivery into the injured tissue is still a biomedical challenge. Gold nanoparticles (GNPs) may be a useful tool for drug delivery into the brain. To evaluate remyelination in the septal nucleus, we administered intracerebral GNPs coupled with EGF (EGF–GNPs). C57BL6/J mice were demyelinated with 0.4% cuprizone (CPZ) and divided into several groups: Sham, Ctrl, GNPs, EGF, and EGF–GNPs. We evaluated the remyelination process at two time-points: 2 weeks and 3 weeks post-injection (WPI) of each treatment. We used the rotarod for evaluating motor coordination. Then, we did a Western blot analysis myelin-associated proteins: CNPase, MAG, MOG, and MBP. EGF–GNPs increase the expression of CNPase, MAG, and MOG at 2 WPI. At 3 WPI, we found that the EGF–GNPs treatment improves motor coordination and increases MAG, MOG, and MBP. EGF–GNPs enhance the expression of myelin-associated proteins and improve the motor coordination in mice. Thus, EGF-associated GNPs may be a promising pharmacological vehicle for delivering long-lasting drugs into the brain.

## 1. Introduction

There are various diseases that affect myelin, for which current treatments are not entirely satisfactory and, therefore, merit the development of new therapeutic alternatives [1,2]. The availability of drugs that stimulate the remyelination process within the brain parenchyma could mitigate the damage caused by demyelinating diseases. However, the efficacy of certain drugs is limited by the difficulty of reaching specific areas of demyelination. In certain circumstances, however, drug delivery vehicles could improve drug delivery to specific areas and prolong the therapeutic effect.

Several studies have shown that epidermal growth factor (EGF) acts as a remyelinating stimulator by promoting the differentiation of oligodendrocyte precursor cells (OPCs) in myelinating oligodendrocytes (OLs), which could be favorable for its application in the treatment of demyelinating diseases [3,4,5,6]. However, its free diffusion within the parenchyma could generate undesirable consequences in the brain cytoarchitecture and neural functioning [7,8]. This drawback could be overcome by the targeted administration of EGF coupled with gold nanoparticles (GNPs) in specific affected regions, where this molecule can act locally and selectively, which would minimize the free dispersion of the drug in the brain parenchyma and, thus, reduce the possible non-toxic effects desired in healthy or unaffected regions. Preliminary studies have shown that GNPs produce a transient response of astrocytes and microglia, which is why we consider that GNPs can be used as a drug depot vehicle and could be an effective means for the targeted administration of this growth factor [9]. To evaluate the remyelinating effect of EGF coupled with nanoparticles, we used a murine model of cuprizone (CPZ) demyelination. The CPZ model is an important tool to evaluate the effectiveness of many drugs [10]. Our data indicated that intracerebral injection of EGF–GNPs improves motor coordination and increases the expression of CNPase and myelin proteins (MAG, MOG, and MBP) in the septal nucleus. These data suggest that EGF coupled with GNPs could be a useful therapeutic tool for the management of demyelinating diseases.

## 2. Materials and Methods

### 2.1. Animals

In this study, 96 mice (male and female) of the C57BL/6J strain of 60 days (postnatal) with a weight of 15–18 g were used. The animals were divided into five groups: intact control group (Ctrl), cuprizone control group (CPZ), nanoparticle-injected group (GNPs), EGF group without nanoparticles vehicle (EGF), and EGF-coupled nanoparticles group (EGF–GNPs). The Ctrl group did not receive any experimental manipulation throughout the study. The rest of the groups underwent demyelination induced with 0.4% CPZ in the diet for 8 weeks. The Ctrl and CPZ groups were subjected to craniotomy without meningeal disruption and did not receive any additional therapeutic manipulation. The GNPs group received a 100 nL intracerebral injection of GNPs without EGF in the septal nucleus. The EGF group received an intracerebral injection of 100 nL EGF (20 ng/100 nL) into the septal nucleus. Lastly, the EGF–GNPs group received an intracerebral injection of 100 nL of EGF–GNPs (20 ng/100 nL) into the septal nucleus. The animals were kept in light–dark cycles of 12:12 h at 22 °C and fed a standard diet for mice (Harlan; crude protein 18.0%, crude fat 5.0%, crude fiber 5%: Cat. 2018S) and water ad libitum. A measure of 0.4% CPZ (SIG-C9012, Sigma, St. Louis, MI, USA) was added to this feed. All the procedures described in this work were approved and supervised by the Ethics Committee for the Care and Handling of Laboratory Animals following the Mexican federal regulations (NOM-ZOO-1999-062).

### 2.2. Cuprizone Administration

To induce demyelination of the brain, mice were fed 0.4% CPZ. First, 500 g of mouse food was ground, then 2 g of CPZ (Cat. SIG-C9012, Sigma) was added to obtain a concentration of 0.4%. Once the mixture was homogenized, we added 400 mL of distilled water and mixed again until we obtained a consistent and uniform mass. Finally, pellets were allowed to dry for 24 h at 40 °C. All pellets were replaced every two days with freshly prepared pellets.

### 2.3. Synthesis of GNPs

GNPs were synthesized using the citrate reduction method from a 1% solution of tetrachloroauric acid (HAuCl_4_, Cat. 27988-77-8, Sigma) and following the protocol described previously [11]. First, 100 mL of deionized water was heated to the boiling point. Then, 1.5 mL of a 1% tetrachloroauric acid solution was added, which was immediately followed by the addition of 5 mL of a 0.05 M sodium citrate solution (Cat. W302600, Sigma). The solution was constantly stirred for 20 min until a ruby-red solution was obtained. Finally, the solution was removed from the heat source, cooled at room temperature, and stored at 4 °C.

### 2.4. EGF–GNPs Nanocomposite

The coupling of EGF with GNPs was performed by following the method previously described by González-Pedroza et al. [11]. First, 1000 µL of the GNPs solution was taken and mixed with 500 µL of HEPES buffer solution (Sigma, Cat. H0887) at 20 mM (pH 7.4). Subsequently, 50 µg of EGF (Cat. 01-102, Millipore, Burlington, MA, USA) was diluted in 500 µL of HEPES buffer. Then, 100 µL of EGF solutions and 400 µL of HEPES were taken and stirred for 20 min. Subsequently, 100 µL of 1% PEG (MW = 20,000, Boehringer Ingelheim, Steinheim, Germany; Cat. 81300, Sigma, St. Louis, MI, USA) was added to avoid adding GNPs and centrifuged at 6000 rpm for 18 min. To obtain the final solution, 1000 µL of supernatant was removed, and the solution was redispersed in 1000 µL of PBS (pH 7.4). Finally, the solution was stored at 4 °C. In the final solution, EGF was at a concentration of (20 ng/100 nL). This dose of EGF has been shown to be effective in inducing the proliferation of oligodendrocyte precursor cells [3,4].

### 2.5. UV–Vis Spectroscopy

To determine whether EGF was coupled with GNPs, we analyzed GNPs and EGF–GNPs solutions with UV–Vis spectroscopy as described previously [11,12]. Briefly, 200 µL of each solution was added to the cell of the UV–Vis equipment (Varian Cary 5000 UV–Vis-NIR, Agilent Technologies, Santa Clara, CA, USA), then each solution was diluted in 800 µL of deionized water until cell was filled. All samples were analyzed in the absorption range of 200–800 nm.

### 2.6. Transmission Electron Microscopy (TEM)

The morphology and size of the GNPs and EGF–GNPs were characterized using a TEM transmission electron microscope (JEOL-2100 200 kV with LaB6 filament; JEOL Ltd. Akishima, Japan). One drop of each solution was placed on a copper grid covered with formvar resin (Sigma, Cat. TEM-FF100CU) and allowed to dry for 20 min before testing at room temperature; the copper grid was subsequently introduced to the sample holder and analyzed. Approximately 1000 nanoparticles were examined and measured with the ImageJ software version 1.52p (NIH; Bethesda, MD, USA) [11,12].

### 2.7. FTIR Analysis

To characterize whether the EGF–GNPs coupling alters the functional groups in the molecule of EGF, we used Fourier transform infrared spectrometry (FTIR; Bruker Tensor, Model 27; Bruker Corporation, Billerica, MA, USA). Briefly, one drop of each solution was added to the test plate (and allowed to dry at room temperature for 20 min), and the equipment was run under standard conditions (transmittance mode, from wavenumber 600 to 4000 cm^−1^). This procedure was performed according to the protocol described previously [11,12].

### 2.8. Administration of EGF–GNPs

A measure of 100 nL of each treatment (GNPs, EGF, and EGF–GNPs) was administered at three points of the septal nucleus using a microinjection with glass needles. The glass needles (tip diameter ~40 µm) and stereotaxic microinjection were carried out as previously described [9,13]. The glass tips were first mounted on stereotaxic equipment (RWD Life Science, San Diego, CA, USA) for subsequent steps. The mice were anesthetized with ketamine (Anesket, PiSA Farmaceutica, Guadalajara, Mexico) and xylazine (Procin, PiSA) at doses of 90 mg/kg of ketamine and 10 mg/kg of xylazine. After anesthetizing the mouse, the hair was removed from the head, and the skin was disinfected with Microdacyn (Laboratorios Sanfer, Mexico City, Mexico). Subsequently, the mouse was attached to the stereotaxic equipment. The following steps were performed under the observation of a surgical microscope (Zeiss Surgical GmbH; ZEISS, Oberkochen, Germany). To begin the surgery, first, an incision was made in the skin from the level of the ears to bregma with a scalpel. To identify the area of interest, the coordinates were entered and identified (point 1: AP = 1 mm, Z = 3.25 mm; point 2: AP = 0.5 mm, Z = 2.75 mm, point 3: AP = 0 mm, Z = 2, 50 mm) in the mouse skull. The coordinates were marked on the mouse skull for later identification. The craniotomy was performed with a drill (Minimite, Model 750; Dremel, Mt. Prospect, IL, USA) and a 0.8 mm bur (Dremel; Mod. 105, Cat. 26150105AE) at the marked coordinates. Subsequently, the needle was introduced into the brain until it reached the septal nucleus, and then 100 nL of the solutions was passed through a microinjector (Mod. MO-10; Narishige Scientific Instrument Lab. Setagaya City, Japan). It is important to mention that the solution was injected at a speed of 0.20 nL/s to avoid tissue damage. The solution was then allowed to diffuse for two minutes. Subsequently, the needle was withdrawn, and the skin tissue was sutured to close the incision. After surgery, the rodents were administered an analgesic (Ketorolac, Cat. 15005-B; Laboratorios Senosiain, Mexico City, Mexico) at a dose of 0.5 mg/kg and housed in a pre-warmed bed at 37 °C until anesthesia recovery.

### 2.9. Rotarod Test

To assess the motor coordination, the fixed-speed rotarod test was performed [14]. This test allows us to evaluate the neurological deficit. Three training tests were carried out for three days (one each day). The training phase consisted of placing the mouse on the rotating drum (3 cm in diameter) at 15 and 25 rpm for 60 s at each speed. The experimental protocol consisted of evaluating the motor coordination of the mouse at 8, 15, 30, and 35 rpm for 60 s for each speed with 5 min breaks in between speeds. For data acquisition, two trials per animal were carried out on the same day at a fixed time (4:30 p.m.), i.e., each mouse received the full behavioral battery twice a day with a 20 min interval between trials. Thus, the mean of fall latencies obtained at every rod speed (8, 15, 30, and 35 rpm) was calculated for each animal. The evaluation was carried out 3 weeks after the intracerebral injection. During the test, no mice were unable or refused to perform the task. Since this test may be biased by individual physical performance and skills, the data obtained for each animal at every rotarod speed (15, 30, and 35 rpm) were normalized with respect to their own data obtained at the lowest speed (8 rpm) [15].

### 2.10. Western Blot

The detection of the myelin proteins CNPase, MAG, MOG, and MBP was carried out with the Western blot technique. To extract the sample, the animals received an anesthetic dose of pentobarbital (50 mg/kg) and were subsequently sacrificed by decapitation, following which the brain was rapidly removed. Once the brain was obtained, it was placed in 0.1 M PBS on ice, and the septal nucleus was dissected and stored at −80 °C. The protein extraction obtained from the septal nucleus was performed using the Total Protein Extraction Kit buffer (Cat. 2140RF; Millipore, Burlington, MA, USA). The collected material was stored at −20 °C and the protein reading of each sample was subsequently carried out on a spectrophotometer (QIAxpert, QIAGE, Hilden, Germany). A measure of 50 µg of protein was used for each sample, which was separated by electrophoresis in polyacrylamide gels (SDS-PAGE) at a concentration of 10% for the detection of MOG (28 kDa), CNPase (47 kDa), and MAG (100 kDa), and at a concentration of 15% for the detection of MBP (12/18 kDa). Protein transfer was performed on polyvinylidene fluoride (PVDF) membranes in a Trans-Blot Turbo Transfer System (Bio-Rad, Hercules, CA, USA) for 30 min. Subsequently, the membranes were incubated in a blocking solution (90% 1X PBS + 0.1% Tween + 10% fetal bovine serum) for 1 h at room temperature, followed by a second blocking solution (10% milk in 1X PBS + Tween 0.1%) for 48 h with shaking and at 4 °C. Then, the primary antibodies β-actin (Santa Cruz Biotechnology, Dallas, TX, USA; Cat. sc-47778; dilution 1:1000), MAG (Cell signaling Technology, Danvers, MA, USA; Cat. 9043; dilution 1:1000), CNPase (Cell signaling, Cat. 5668), MOG (Santa Cruz, Cat. sc-73330; dilution 1:1000), and MBP (Cell signaling, Cat. 78896F; dilution 1:1000) were added and allowed to incubate for 24 h at 4 °C. The next day, the membranes were washed eightfold (1X PBS + 0.1% Tween) for 5 min under shaking. Following this, they were incubated with a secondary antibody anti-mouse (M-IgGκ-BP-HRP, Cat. sc-516102; Santa Cruz Biotechnology, Dallas, TX, USA) for β-actin and MOG, and anti-rabbit (mouse anti-rabbit IgG-HRP, Santa Cruz, Cat. sc-2357) for MAG, CNPase, and MBP for 2 h at room temperature and with shaking. To remove the excess of secondary antibody, eight 5 min washes were administered. Finally, the chromogenic solution (Thermo Fisher Scientific, Waltham, MA, USA; 1-Step™ Ultra TMB, Cat. 37574) was added to reveal the brand and revealed until an appropriate detection level of proteins was observed. The bands were analyzed in the ImageJ software (NIH, 1.52p) to obtain the number of pixels on each detected band. To determine the amount of each protein, the pixels of each labeled band from each subject were normalized with their respective β-actin expression and relativized with the control group. We utilized 3 mice per group for each timepoint (2 WPI and 3 WPI), and each experiment was performed by duplicate. Then, we calculated the average of both measurements per mouse, and the statistical analysis and plots were performed with these mean values. Thus, the sample size (*n* = 3 animals per group) also represented the number of data analyzed per group.

### 2.11. Statistical Analysis

For the analysis of the data obtained with the behavioral tests, the multivariate non-parametric Kruskal–Wallis test followed by Bonferroni correction was performed and point-and-line graphs were used to display these data. The data were expressed with the mean and standard error (SE). The Mann–Whitney *U* test was used for Western blot analysis. All data were expressed as the mean and standard error and represented through bar graphs. The significant probability was established with values of *p* ≤ 0.05. For the statistical analysis of multiple correlation between the groups, the SPSS version 21 program (IBM, Chicago, IL, USA) was used. The graphs were made in GraphPad Prism version 8 (GraphPad Software Inc., San Diego, CA, USA).

## 3. Results

### 3.1. UV–Vis Analysis of EGF–GNPs

**Figure 1:**
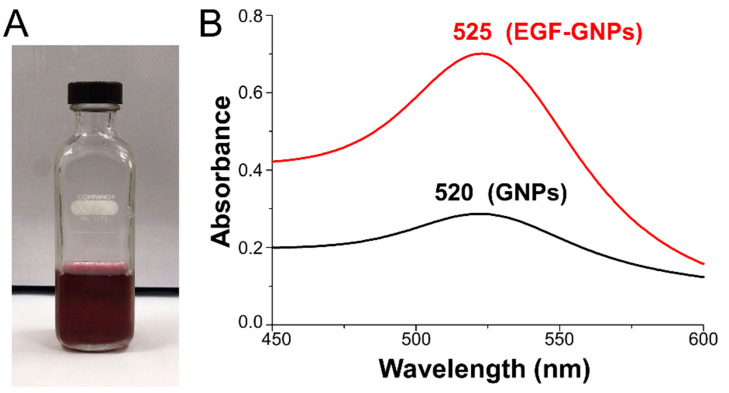
Synthesis, optical properties, and elemental mapping of GNPs and EGF–GNPs. (**A**) Representative ruby-red solution of gold nanoparticles obtained by citrate reduction method. (**B**) UV–Vis spectra, the black peak at 520 nm corresponds to GNPs and the red peak at 525 nm corresponds to the nanocomposite.

To couple EGF with GNPs, we used the citrate reduction method as described previously [16], and a typical ruby-red solution was obtained (Figure 1A). In the UV–Vis analysis, a maximum absorption peak of 520 nm was observed for the GNPs, whereas the nanocomposite (EGF–GNPs) showed a single peak of 525 nm (Figure 1B), which suggest that EGF and GNPs conformed to a uniform molecular aggregate.

### 3.2. Structural Analysis of EGF–GNPs

**Figure 2:**
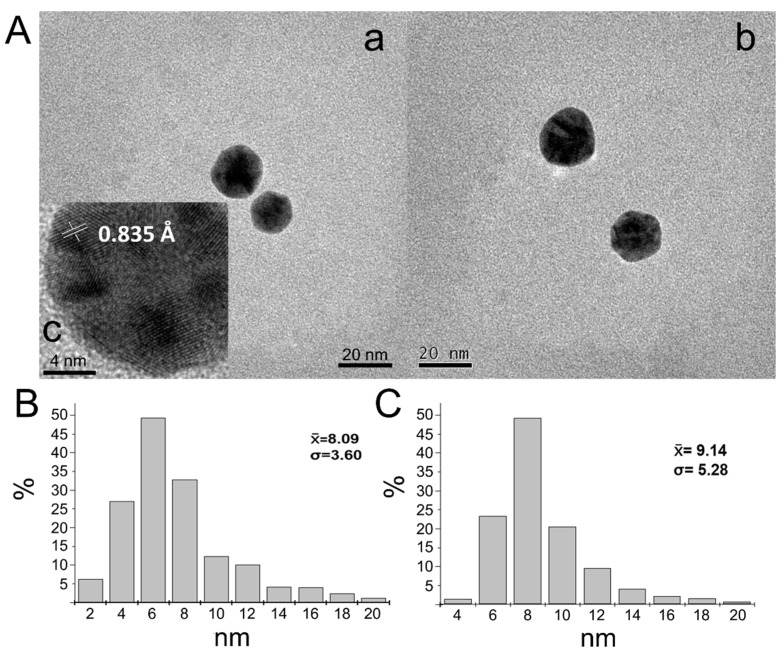
Morphological analysis of GNPs and EGF–GNPs. (**A**) TEM analysis shows nanoparticles have aspherical morphology in both naked GNPs (**a**) and EGF–GNPs (**b**). High-resolution TEM (HR-TEM) reveals an interplanar distance of 0.835 Å; (**c**) histograms show an average size of 8.09 ± 3.60 and 9.14 ± 5.28 for GNPs (**B**) and EGF–GNPs (**C**), respectively. Scale bars = 20 nm (**a**,**b**) and 4 nm (**c**).

Non-spherical morphologies may represent an inconvenience for administrating nanoparticles into a tissue [17]. Furthermore, GNPs are chemically inert when their sizes are larger than 3 nm [18]. To determine the size and morphology of our GNPs as well as that of the EGF–GNPs, we used TEM. In all the samples analyzed, we observed that our GNPs had a spherical morphology (Figure 2A). The mean size of the uncoupled nanoparticles was 8.09 ± 3.60 nm and that of the EGF–GNPs nanocomposite was 9.14 ± 5.28 nm in diameter (Figure 2B,C). The interplanar distance of GNPs was 0.835 Å (Figure 2A, insert c), which indicates that GNPs have a crystalline structure typically found in gold-containing solutions. These findings indicate that GNPs are chemically inert and have a spherical morphology.

### 3.3. FTIR Analysis of EGF–GNPs

**Figure 3:**
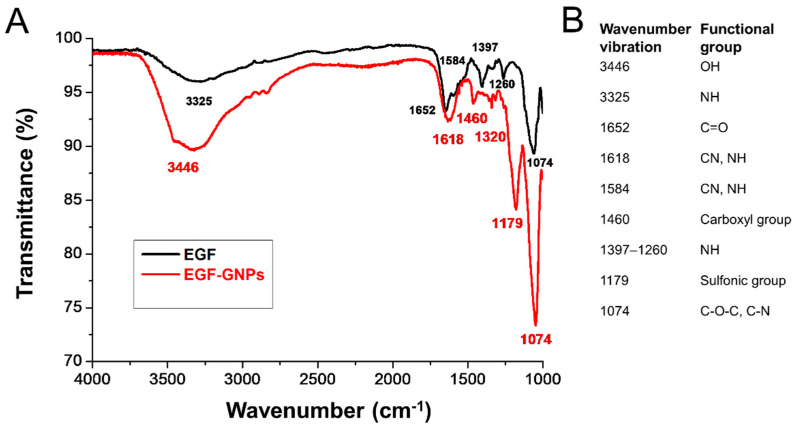
FTIR spectra of GNPs and EGF–GNPs. (**A**) The black band in the spectra correspond to the EGF molecule alone, whereas the red band correspond to the EGF molecule coupled to GNPs. The black band of the EGF shows the typical spectrum for this protein. The attachment of the EGF to GNPs by electrostatic interactions did not affect the functional groups of the EGF molecule (red band). (**B**) Summarized information of each wavenumber vibration per functional group.

To determine that the molecule of EGF is not affected by the coupling with GNPs, we carried out a FTIR analysis. In all our samples, we found nine absorption bands in the infrared spectrum, a physicochemical characteristic of EGF [19,20] (Figure 3). The black band in the infrared spectrum corresponds to the uncoupled EGF (Figure 3A); each wavenumber vibration in the spectrum represents a functional group of the EGF. The red band in the infrared spectrum corresponds to the EGF coupled to the GNPs (Figure 3A). Our data indicate that EGF alone and EGF–GNPs showed the same functional groups. This evidence indicates that EGF molecules are not affected by the coupling with GNPs.

### 3.4. Body Weight Gain

**Figure 4:**
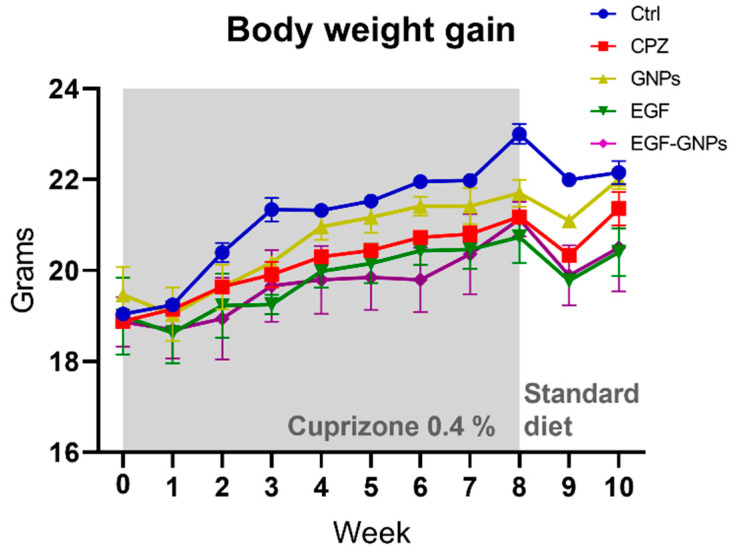
Body weight gain. As expected, the control group (Ctrl) showed higher body weight gain than the CPZ-fed animals. One week after the surgical manipulation, all animals show a slight decreased in body weight. In subsequent weeks, all groups start recovering their body weight, and no significant differences were observed among groups at the end of the study. Data are expressed as the mean ± SE. Ctrl (*n* = 21), CPZ (*n* = 22), GNPs (*n* = 17), EGF (*n* = 17), and EGF–GNPs (*n* = 19).

To promote demyelination, the mice were fed standard food supplemented with 0.4% cuprizone for 8 weeks, which produces a slight reduction in the weight gain during the intoxication period [21,22,23]. Thus, we monitored the weight gain in all our animals every week throughout the study. Our data showed that the Ctrl group increased their body weight by 20% at week 8, while the rest of the CPZ-treated groups showed an increase of around 11% with respect to week 0 (Figure 4). All groups show a moderate weight reduction in the first week after cuprizone, which could be due to the surgical procedure; however, all of them recovered their body weight and reached levels similar to the Ctrl group in the following weeks (Figure 4).

### 3.5. EGF–GNPs Improves Motor Coordination

**Figure 5:**
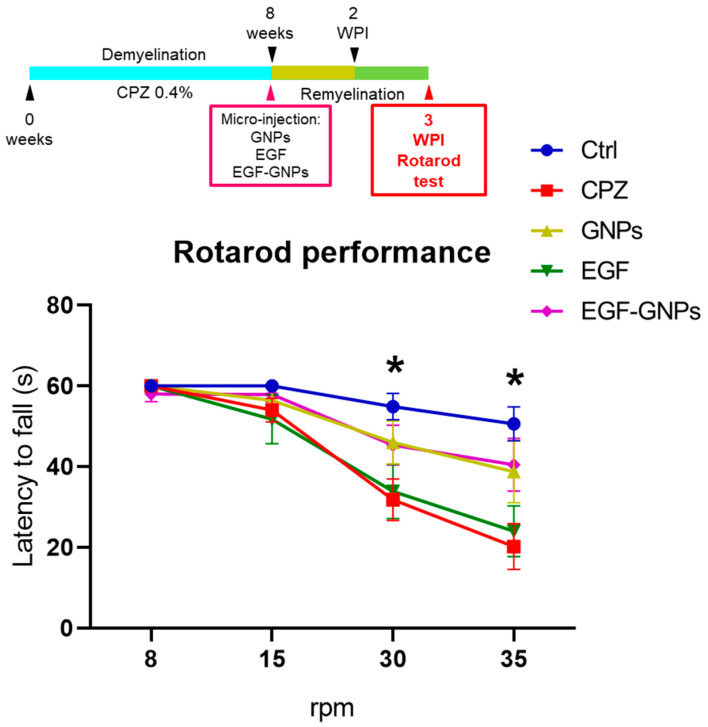
Motor coordination. Motor coordination was assessed at 3 WPI. Differences in motor performance were more evident with the rotor speeds of 30 and 35 rpm. Ctrl (*n* = 10), CPZ (*n* = 12), GNPs (*n* = 8), EGF (*n* = 8) and EGF–GNPs (*n* = 8). Data are expressed as the mean ± SE. * *p* ≤ 0.05; Kruskal–Wallis test with Bonferroni correction. At 30 rpm speed, asterisks indicate differences between the Ctrl vs. CPZ (*p* = 0.003) and EGF (*p* = 0.010). At 35 rpm speed, Ctrl vs. CPZ (*p* = 0.001) and EGF (*p* = 0.005).

CPZ removal allows spontaneous and complete remyelination in around 6 weeks [10]. Thus, we assessed the effect of intracerebral treatments on motor coordination during the early stages of this period. Three weeks after the GNPs micro-injections (3 WPI), we applied the rotarod test. We evaluated the motor performance of all groups using four different rotational speeds (8, 15, 30, and 35 rpm), as shown in Figure 5. No statistically significant differences among all the groups were found at speeds of 8 rpm (*H* = 0.314) and 15 rpm (*H* = 0.137). At 30 rpm, we found statistically significant differences among the groups (*H* = 0.026). Thus, the Ctrl group (54.9 ± 3.2 s) showed better performance than the untreated CPZ group (31.8 ± 5.1 s, *p* = 0.003) and the uncoupled EGF group (33.8 ± 6.7 s, *p* = 0.01). At 35 rpm (*H* = 0.006), the best performance was observed in the Ctrl group (50.6 ± 4.2 s) as compared to the CPZ (20.2 ± 5.6, *p* = 0.001) and the EGF group (24.1 ± 6.3, *p* = 0.005). We did not find significant differences in the EGF–GNPs group when compared to the other groups in all the evaluated speeds; however, the statistical tendency was seen when compared with the CPZ and EGF groups. See Supplementary Tables S1–S3 for statistics. These findings suggest that motor deficits are more evident in the cuprizone-treated animals as the rotational speed increases and that the treatments with EGF–GNPs and GNPs show a tendency to improve the motor performance of animals.

### 3.6. EGF–GNPs Increase CNPase, MAG, MOG and MBP Proteins

**Figure 6:**
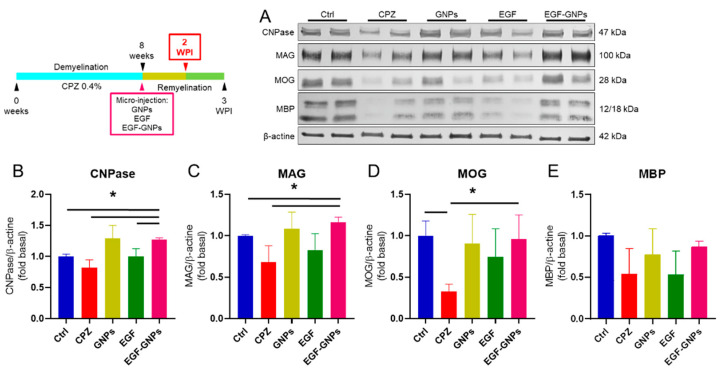
Myelin protein expression in the septal nucleus at 2 weeks post-injection. (**A**) Two representative images of Western blot of myelin-associated protein expressions per group. (**B**) CNPase expression was increased in the EGF–GNPs group with respect to the Ctrl, CPZ, and EGF groups. (**C**) MAG expression was also elevated in the EGF–GNPs group compared to the Ctrl and CPZ groups. (**D**) MOG expression show differences between EGF–GNPs vs. GNPs group and Ctrl vs. GNPs group. (**E**) MBP expression did not show statistically significant differences among all groups. All procedures were processed by duplicate; *n* = 3 mice per group. Data are expressed as the mean ± SE. Mann–Whitney *U* test, * *p* ≤ 0.05.

**Figure 7:**
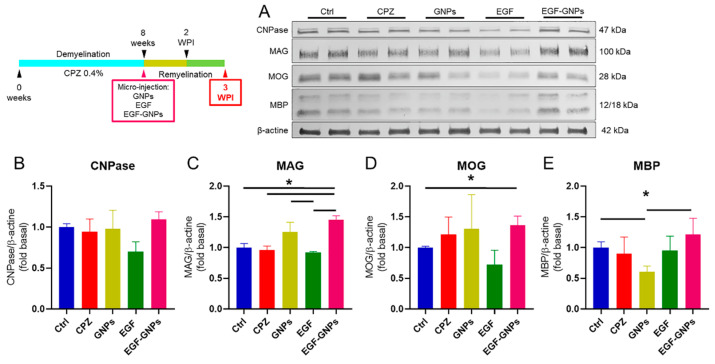
Myelin protein expression in the septal nucleus at 3 weeks post-injection. (**A**) Two representative images of Western blot of myelin-associated proteins expression per group. (**B**) CNPase expression did not show statistically significant differences among all groups. (**C**) MAG expression remains elevated in the EGF–GNPs group as compared to the Ctrl, CPZ, and EGF groups. (**D**) MOG expression is increased in the EGF–GNPs group compared to the Ctrl group. (**E**) MBP expression show differences between GNPs group compared to the Ctrl and EGF–GNPs groups. All procedures were processed by duplicate; *n* = 3 mice per group. Data are expressed as the mean ± SE. Mann–Whitney *U* test, * *p* ≤ 0.05.

We evaluated the expression of the enzyme CNPase and the myelin proteins MAG, MOG, and MBP in the septal nucleus by Western blot at two timepoints: two weeks post-injection (2 WPI) and three weeks post-injection (3 WPI). At the 2 WPI, our results indicated that the CNPase protein increased in the EGF–GNPs (1.26 ± 0.02) group when compared to the Ctrl (1.00 ± 0.03, *U* = 0, *p* = 0.05), CPZ (0.81 ± 0.12, *U* = 0, *p* = 0.05), and EGF (0.99 ± 0.12, *U* = 0, *p* = 0.05) groups (Figure 6B). At 3 WPI, the expression of CNPase did not show significant changes among all groups (Figure 7B), suggesting that this enzyme decreases as the remyelination process progresses. The analysis of MAG at 2 WPI indicated statistically significant differences between the EGF–GNPs (1.16 ± 0.05) group with respect to the Ctrl (1.00 ± 0.01, *U* = 0, *p* = 0.05) and CPZ (0.82 ± 0.19, *U* = 0, *p* = 0.05) groups (Figure 6C). Interestingly, at week 3, the differences in MAG expression became more evident when comparing the EGF–GNPs (1.44 ± 0.06) group against the CPZ (0.96 ± 0.06, *U* = 0, *p* = 0.05), Ctrl (1.00 ± 0.06, *U* = 0, *p* = 0.05), and EGF groups (0.92 ± 0.13, *U* = 0, *p* = 0.05) (Figure 7C). Similarly, we found statistically significant differences between the GNPs (1.25 ± 0.15) and the EGF group (0.92 ± 0.13, *U* = 0, *p* = 0.05).

At 2 WPI, MOG protein shows statistically significant low levels in the CPZ (0.32 ± 0.08) group (the untreated group) as compared to the intact control group (Ctrl) (1 ± 0.17, *U* = 0, *p* = 0.05) and the EGF–GNPs (0.95 ± 0.29, *U* = 0, *p* = 0.05) group (Figure 6D). At 3 WPI, MOG levels in the EGF–GNPs (0.91 ± 0.09) group increased significantly when compared to the Ctrl (1 ± 0.02, *U* = 0, *p* = 0.05) group (Figure 7D). For MBP, we did not find statistical differences at 2 WPI (Figure 6E). However, at 3 WPI, the analysis of the MBP protein showed statistically significant differences between the nanocomposite EGF–GNPs (1.21 ± 0.26) and Ctrl (1 ± 0.09) group vs. the GNPs (0.61 ± 0.08, *U* = 0, *p* = 0.05) group (Figure 7E), which indicates that EGF coupled with GNPs favored the expression of MBP until reaching levels very similar to the intact control group. Similarly, the GNPs group showed similar protein levels with the EGF–GNPs groups at both timepoints. At 2 WPI, CNPase: the GNPs (1.20 ± 0.2) vs. the EGF–GNPs group (1.26 ± 0.02; *U* = 3, *p* = 0.513); MAG: the GNPs (1.08 ± 0.19) vs. the EGF–GNPs group (1.16 ± 0.05; *U* = 3, *p* = 0.513); MOG: the GNPs (0.90 ± 0.35) vs. the EGF–GNPs group (0.95 ± 0.29; *U* = 4, *p* = 0.827); MBP: the GNPs (0.77 ± 0.31) vs. the EGF–GNPs group (0.86 ± 0.06; *U* = 3, *p* = 0.513). At 3 WPI, CNPase: the GNPs (0.97 ± 0.22) vs. the EGF–GNPs group (1.09 ± 0.09; *U* = 3, *p* = 0.513); MAG: the GNPs (1.25 ± 0.15) vs. the EGF–GNPs group (1.44 ± 0.06; *U* = 2, *p* = 0.275); MOG: the GNPs (1.30 ± 0.55) vs. the EGF–GNPs group (1.36 ± 0.14; *U* = 3, *p* = 0.513). All data and summary statistics are shown in Supplementary Tables S3 and S4. For blots images, please see Supplementary Figures S1 and S2. In summary, we observed a significant increase in the levels of protein CNPase, MAG, MOG and MBP in the EGF–GNPs group as compared to the untreated groups. These findings suggest that EGF coupled with GNPs accelerates or facilitates the remyelination process.

## 4. Discussion

In the present study, we analyzed the effect of the nanocomposite EGF–GNPs, which could act as a depot formulation to have a sustained and local response that may prevent the free dispersal of EGF within the brain parenchyma. First, we built the EGF–GNPs nanocomposite and delivered it into the brains of cuprizone-demyelinated mice. Our findings indicate that EGF–GNPs nanocomposite was effective in promoting motor strength and coordination. Subsequently, we analyzed the expression of proteins related to the remyelination process of CNPase, MAG, MOG, and MBP in the septal nucleus. This region was chosen because it has a significant number of myelinated axonal tracts; furthermore, due to its size and location, it is a very accessible target for brain microinjections. Our data indicated that EGF–GNPs significantly increased the levels of CNPase, MAG, MOG, and MBP in the septal nucleus, suggesting that the coupling of EGF with GNPs favors the myelin regeneration process.

### 4.1. Synthesis and Characterization of the Nanocomposite

For the synthesis of the nanocomposite, we first made the synthesis of GNPs using the citrate reduction method, to which we later coupled the EGF and PEG, as previously described [11]. Colorimetric observations indirectly suggested the presence of nanoparticles with spherical morphology, and UV–Vis analysis showed the presence of a single plasmon resonance peak for the GNPs at 520 nm and a similar peak, at 525 nm, for the EGF-coupled GNPs; this displacement in the band is expected as a consequence of the attachment of the EGF to the GNPs [11]. This characteristic is also suggestive of a spherical morphology of the nanoparticle because two peaks in the spectrum are strongly suggestive of cylindric or rod-type morphologies [24,25]. To corroborate this assertion, we analyzed the nanoparticles using TEM; hence, we were able to observe the spherical morphology of the nanoparticles. Obtaining and maintaining the spherical morphology in a nanoparticle is of great importance since previous studies have shown that spherical GNPs cause limited glial reactivity in vivo [9] compared to other morphological conformations such as rods or stars [17]. The analysis with FTIR showed the typical infrared spectrum for EGF [26,27]. Vibrational changes in the functional groups of the EGF in the nanocomposite suggest that the coupling with EGF was successful [11]. This evidence allowed us to establish that our method of synthesizing the nanocomposites was consistent and fulfilled the physicochemical characteristics necessary for use in our subsequent experiments.

### 4.2. Body Weight Gain

To promote demyelination, we used a known administration model of 0.4% cuprizone mixed with standard food [10]. Subsequently, the body weight gain was monitored every week for 8 weeks. In our study, we found that the mice that did not receive cuprizone in their food had a greater weight gain, compared to those who were treated with 0.4% cuprizone. Moderate weight loss is a common feature in the cuprizone model, which in no case leads to malnutrition or the general development of the subjects being compromised, as previously reported in other studies [21,22,23].

### 4.3. The Nanocomposite EGF–GNPs Improves Motor Coordination at 3 Weeks Post-Injection

To determine whether EGF–GNPs produced any effect on motor coordination, we used the rotating rod test. Previous studies have shown that mice treated with cuprizone decrease their performance in the apparatus, showing a greater latency to fall [28,29]. The latter agrees with our findings at week 3 post-injection, where we found better performance of the rotating rod in the Ctrl group at 15, 30, and 35 rpm speeds. We also observed that the EGF–GNPs and GNPs groups showed a tendency to perform better than the other groups. This would indicate that EGF–GNPs and the GNPs could have a therapeutic effect that favors functional recovery. As mentioned above, the soluble EGF group had a lower performance compared to the control, GNPs, and EGF–GNPs groups. These findings once again highlight the importance of GNPs as a pharmacological vehicle, as it shows that EGF bound to nanoparticles improves motor coordination. The biological mechanism by which the EGF–GNPs improves the motor coordination may be by promoting the expression of myelin proteins.

### 4.4. The EGF–GNPs Promotes the Differential Expression of Myelin Proteins

The formation of the myelin sheath is the result of myelination and remyelination and, in both cases, this occurs when the cytoplasmic processes of pre-myelinating oligodendrocytes (OLs) encounter the axons of the neuron. To quantify the changes in myelin that occur in the septal nucleus during the remyelination phase, we performed the Western blot technique to detect the CNPase enzyme and the MAG, MOG, and MBP proteins. At week 2 WPI, we found that CNPase is more abundant in the EGF–GNPS group with respect to the Ctrl, EGF, and CPZ groups, but at week 3 WPI, the levels of CNPase are similar in all groups. CNPase is an enzyme that is present in the early stages of remyelination and is expressed by pre-myelinating OLs [30,31,32,33]. Therefore, the increase in CNPase in the EGF–GNPs group suggests a greater presence of pre-myelinating OLs in the early stages of remyelination, which may eventually decrease after 3 weeks of recovery, representing an expected and desirable phenomenon during events of remyelination [33]. In the first timepoint, which we evaluated 2 WPI, the MOG protein is elevated in the EGF–GNPs group compared to the CPZ group, and its numerical value was significantly similar to that of the Ctrl group; therefore, we can infer that EGF–GNPs promote the normalization of MOG levels. For the second timepoint (3 WPI), the MOG levels in the EGF–GNPs group remain high compared to the rest of the groups; thus, we can deduce that the EGF–GNPs promote an increase in MOG by a longer period.

On the other hand, the MAG analysis showed similar data to MOG. At 2 WPI, the group administered with EGF–GNPs showed an increase in MAG, compared to the Ctrl and CPZ groups, which suggest that an early remyelination is ongoing [34,35,36]. At 3 WPI, the increase in MAG was more evident in the EGF–GNPs group with respect to the CPZ and EGF groups, indicating that soluble EGF (not coupled with GNPs) is less effective in promoting myelin protein synthesis than the EGF coupled with GNPs. This phenomenon could be caused by a nanoparticle-mediated “anchoring” system, which would promote a local response of EGF by not allowing its free dispersion and by functioning as a drug depot vehicle. The latter further reinforces the relevance of this system; since EGF is highly mitogenic, its free dispersal in the brain could have unintended consequences. Regarding the MBP analysis, the CPZ, GNPs, and EGF groups show a slight reduction compared to the Ctrl and EGF–GNPs groups; however, the data indicate that statistically significant differences become more apparent at 3 WPI, where the EGF–GNPs group shows a significant increase compared to the control vehicle group (GNPs).

**Figure 8:**
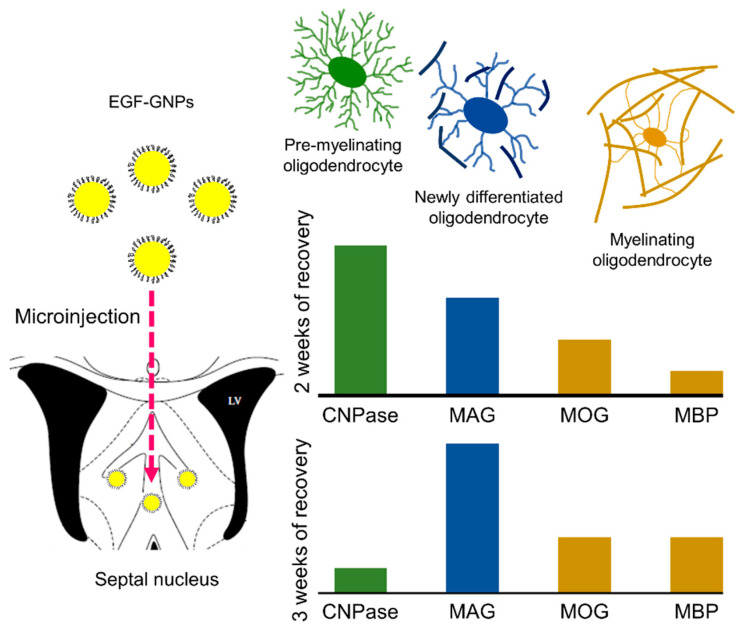
Representative scheme of the EGF–GNPs effect on the expression of myelin-associated proteins in the septal nucleus. Two weeks after the injection of EGF–GNPs, the most abundant protein is CNPase, a marker of pre-myelinating OLs, followed by MAG (a marker of newly differentiated OLs) and MOG (marker of myelinating OLs). At the 3rd week after EGF–GNPs administration, CNPase levels tend to normalize, whereas the MAG, MOG, and MBP expression increases. These findings suggest that EGF–GNPs facilitates the remyelination process.

EGF is a mitogenic protein that promotes different processes such as survival, cell division, differentiation, proliferation, and migration of different cell types [37,38]. In the CNS, EGF promotes the glial lineage and, particularly, the oligodendrocyte lineage [3,4,6,39]. In addition, it favors the conservation of myelin and its synthesis through stimulation with EGF [4,5,40]. Existing studies evaluating the direct effect of EGF on OPCs both in vitro and in vivo are scarce. Indirectly, EGF has been shown to stimulate IGF-1 release from astrocytes, which in turn stimulates myelination [41]. In in vitro demyelination models, EGF has also been observed to promote the remyelination and expression of MBP and CNPase [42] and appears to be more efficient for this process than other growth factors such as FGF and PGDF [43]. In fact, the downregulation of EGFR (in vivo) in OPC NG2+ reduces the maturation process in myelinating OLs [44]. Thus, growing evidence indicates that EGF actively promotes remyelination and OL production in the postnatal brain. However, this molecule produces a massive proliferation and migration of OPC to the brain that significantly affects the cellular composition and cytoarchitecture of various brain regions [3]. Intriguingly, the GNPs and EGF–GNPs groups showed similar performance in the rotarod test and myelin-related proteins (except for MBP) at both timepoints (2 WPI and 3 WPI). These phenomena may be explained by the transient activation of astrocytes and microglia cells produced when GNPs are injected into the brain parenchyma [9]. Glial activation is the main source of cytokines and growth factors, including EGF, which promotes brain repair and regeneration [45]. Thus, chemical mediators produced by reactive astrocytes and microglia cells can stimulate endogenous progenitors that, in turn, accelerate myelin repair [41]. Remarkably, the injection of vehicle solution and uncoupled EGF did not produce these beneficial effects, which suggests that the injection injury or a small dose of EGF by themselves were not enough to trigger a restorative response in the white matter. However, further research is required to confirm this hypothesis. In summary, our findings indicate that gold nanoparticles functionalized with EGF can accelerate the remyelination process by promoting the expression of myelin-associated proteins during the early stages of remyelination (CNPase and MAG) and the subsequent expression of MOG and MBP at later stages (Figure 8). Our study shows that nanoparticles can function as a pharmacological vehicle for EGF, which reduces its free dispersion in the brain parenchyma. However, more studies are required to explore the effects of EGF–GNPs on brain cytoarchitecture to confirm or reject this possibility.

### 4.5. Limitations of the Study

In our study, the reduced sample size in the Western blot analysis can affect the statistical power and some interpretations of these results. Moreover, the method of adjustment for multiple comparisons cannot be applied with small sample sizes. Thus, these data may be considered as exploratory-type findings, and further research is needed to confirm some of these interpretations.

## 5. Conclusions

EGF–GNPs facilitate the remyelination of the septal nucleus by increasing the expression of CNPase and the myelin proteins MAG, MOG, and MBP. These molecular changes induced by EGF–GNPs improve motor coordination. Therefore, our results suggest that EGF coupled with GNPs could be a viable alternative for promoting and accelerating the remyelination process.

## Data Availability

Not applicable.

## Supplementary Materials

The following supporting information can be downloaded at: https://www.mdpi.com/article/10.3390/life12030333/s1, Table S1: Descriptive statistics for rotarod, latency to fall; Table S2: Rotarod test performance, statistical analysis for 3 WPI, Kruskal-Wallis H test; Table S3: Rotarod test performance, statistical analysis for 3 WPI, Bonferroni correction for pair comparison; Table S4: Descriptive statistics for Western blot. Table S5: Myelin proteins, statistical analysis for 2 WPI and 3 WPI, Mann-Whitney *U* test. Figure S1: Blots obtained at 2 weeks of recovery. Figure S2: Blots obtained at 3 weeks of recovery.
