# Supplementary material for: EGF-Coupled Gold Nanoparticles Increase the Expression of CNPase and the Myelin-Associated Proteins MAG, MOG, and MBP in the Septal Nucleus Demyelinated by Cuprizone
Source: Life (Basel). 2022 Feb 23;12(3):333. doi: 10.3390/life12030333 (PMC8955175; doi:10.3390/life12030333)
Supplement: Supplementary file 1 [file life-12-00333-s001.zip › life-1606637-supplementary.pdf]

life

MDPI

Supplementary

EGF-Coupled Gold Nanoparticles Increase the Expression of CNPase and the Myelin-Associated Proteins MAG, MOG, and MBP in the Septal Nucleus Demyelinated by Cuprizone

Eduardo Lira-Diaz [1,2], Jesus Monroy-Rodriguez [3], Maria G. Gonzalez-Pedroza [4], Raul A. Morales-Luckie [5], Luis Castro-Sánchez [3,6] and Oscar Gonzalez-Perez [1,*]

1 Laboratory of Neuroscience, School of Psychology, University of Colima, Colima 28040, Mexico; elira@ucol.mx and osglez@ucol.mx
2 Physiological Science PhD Program, School of Medicine, University of Colima, Colima 28040, Mexico
3 University Center for Biomedical Research, University of Colima, Colima 28045, Mexico; jmonroy@ucol.mx (J.M.-R.); luis_castro@ucol.mx (L.C.-S.)
4 Department of Biotechnology, School of Sciences, Autonomous University of the State of Mexico, Toluca 50200, Mexico; mggonzalezp@uaemex.mx
5 Department of Nanomaterials, Joint Center for Research in Sustainable Chemistry UAEMex—UNAM (CCIQS), Autonomous University of the State of Mexico (UAEMex), Toluca 50200, Mexico rmoralesl@uaemex.mx
6 CONACyT-University of Colima, Colima 28045, Mexico
* Correspondence: osglez@ucol.mx

Table S1. Descriptive statistics for rotarod, latency to fall.

8 rpm		15 rpm	
**Group**	**Mean ± SE (seconds)**	**Group**	**Mean ± SE (seconds)**
Ctrl	60 ± 0	Ctrl	60 ± 0
CPZ	60 ± 0	CPZ	54 ± 2.94
GNPs	60 ± 0	GNPs	56.43 ± 3.14
EGF	60 ± 0	EGF	51.75 ± 6.09
EGF-GNPs	58.06 ± 1.93	EGF-GNPs	57.87 ± 1.91
**30 rpm**		**35 rpm**	
**Group**	**Mean ± SE (seconds)**	**Group**	**Mean ± SE (seconds)**
Ctrl	54.9 ± 3.25	Ctrl	50.6 ± 4.23
CPZ	31.83 ± 5.12	CPZ	20.20 ± 5.68
GNPs	46 ± 5.36	GNPs	38.75 ± 7.70
EGF	33.87 ± 6.75	EGF	24.06 ± 6.30
EGF-GNPs	45.37 ± 4.96	EGF-GNPs	40.5 ± 6.51

Table S2. Rotarod test performance, statistical analysis for 3 WPI, Kruskal-Wallis H test.

Speed	Kruskal-Wallis test
8 rpm	$H = 0.314$
15 rpm	$H = 0.137$
30 rpm	$\mathbf{H = 0.026}$*
35 rpm	$\mathbf{H = 0.006}$*

Table S3. Rotarod test performance, statistical analysis for 3 WPI, Bonferroni correction for pair comparison.

Comparison	30 rpm	35 rpm
	***p* value**	***p* value**

Ctrl vs CPZ	***p* = 0.003***	***p* = 0.001***
Ctrl vs GNPs	*p* = 0.235	*p* = 0.244
Ctrl vs EGF	***p* = 0.010***	***p* = 0.005***
Ctrl vs EGF-GNPs	*p* = 0.267	*p* = 0.248
CPZ vs GNPs	*p* = 0.131	*p* = 0.055
CPZ vs EGF	*p* = 0.934	*p* = 0.840
CPZ vs EGF-GNPs	*p* = 0.111	*p* = 0.053
GNPs vs EGF	*p* = 0.192	*p* = 0.116
GNPs vs EGF-GNPs	*p* = 0.940	*p* = 0.993
EGF vs EGF-GNPs	*p* = 0.168	*p* = 0.114

Table S4. Descriptive statistics for Western blot.

**2 weeks post-injection**		**3 weeks post-injection**	
**CNPase**		**CNPase**	
**Group**	**Mean ± SE**	**Group**	**Mean ± SE**
Ctrl	1.00 ± 0.03	Ctrl	1.00 ± 0.04
CPZ	0.81 ± 0.12	CPZ	0.94 ± 0.15
GNPs	1.29 ± 0.20	GNPs	0.97 ± 0.22
EGF	0.99 ± 0.12	EGF	0.69 ± 0.12
EGF-GNPs	1.26 ± 0.02	EGF-GNPs	1.09 ± 0.09
**MAG**		**MAG**	
**Group**	**Mean ± SE**	**Group**	**Mean ± SE**
Ctrl	1.00 ± 0.01	Ctrl	1.00 ± 0.06
CPZ	0.68 ± 0.19	CPZ	0.96 ± 0.06
GNPs	1.08 ± 0.19	GNPs	1.25 ± 0.15
EGF	0.82 ± 0.19	EGF	0.92 ± 0.13
EGF-GNPs	1.16 ± 0.05	EGF-GNPs	1.44 ± 0.06
**MOG**		**MOG**	
**Group**	**Mean ± SE**	**Group**	**Mean ± SE**
Ctrl	1.00 ± 0.17	Ctrl	1.00 ± 0.02
CPZ	0.32 ± 0.08	CPZ	1.21 ± 0.28
GNPs	0.90 ± 0.35	GNPs	1.30 ± 0.55
EGF	0.74 ± 0.33	EGF	0.72 ± 0.23
EGF-GNPs	0.95 ± 0.29	EGF-GNPs	1.36 ± 0.14
**MBP**		**MBP**	
**Group**	**Mean ± SE**	**Group**	**Mean ± SE**
Ctrl	1.00 ± 0.03	Ctrl	1.00 ± 0.09
CPZ	0.53 ± 0.32	CPZ	0.90 ± 0.27
GNPs	0.77 ± 0.31	GNPs	0.61 ± 0.08
EGF	0.53 ± 0.28	EGF	0.95 ± 0.23
EGF-GNPs	0.86 ± 0.06	EGF-GNPs	1.21 ± 0.26

Table S5. Myelin proteins, statistical analysis for 2 WPI and 3 WPI, Mann-Whitney U test.

**2 WPI**	**CNPase**		**MAG**		**MOG**		**MBP**	
**Comparison**	**"*U*" value**	***p* value**	**"*U*" value**	***p* value**	**"*U*" value**	***p* value**	**"*U*" value**	***p* value**
Ctrl vs CPZ	*U* = 3.000	*p* = 0.513	*U* = 2.000	*p* = 0.275	*U* = 0.000	***p* = 0.050***	*U* = 3.000	*p* = 0.513

Ctrl vs GNPs	$U = 2.000$	$p = 0.275$	$U = 3.000$	$p = 0.513$	$U = 4.000$	$p = 0.827$	$U = 3.000$	$p = 0.513$
Ctrl vs EGF	$U = 3.000$	$p = 0.513$	$U = 3.000$	$p = 0.513$	$U = 3.000$	$p = 0.513$	$U = 3.000$	$p = 0.513$
Ctrl vs EGF-GNPs	$U = 0.000$	**$p = 0.050$***	$U = 0.000$	**$p = 0.050$***	$U = 4.000$	$p = 0.827$	$U = 1.000$	$p = 0.127$
CPZ vs GNPs	$U = 1.000$	$p = 0.127$	$U = 2.000$	$p = 0.275$	$U = 2.000$	$p = 0.275$	$U = 2.000$	$p = 0.275$
CPZ vs EGF	$U = 2.000$	$p = 0.275$	$U = 3.000$	$p = 0.513$	$U = 2.000$	$p = 0.275$	$U = 4.000$	$p = 0.827$
CPZ vs EGF-GNPs	$U = 0.000$	**$p = 0.050$***	$U = 0.000$	**$p = 0.050$***	$U = 0.000$	**$p = 0.050$***	$U = 3.000$	$p = 0.513$
GNPs vs EGF	$U = 2.000$	$p = 0.275$	$U = 3.000$	$p = 0.513$	$U = 4.000$	$p = 0.827$	$U = 2.000$	$p = 0.275$
GNPs vs EGF-GNPs	$U = 3.000$	$p = 0.513$	$U = 3.000$	$p = 0.513$	$U = 4.000$	$p = 0.827$	$U = 3.000$	$p = 0.513$
EGF vs EGF-GNPs	$U = 0.000$	**$p = 0.050$***	$U = 1.000$	$p = 0.127$	$U = 2.000$	$p = 0.275$	$U = 3.000$	$p = 0.513$
**3 WPI**	**CNPase**		**MAG**		**MOG**		**MBP**	
**Comparison**	**"*U*" value**	***p* value**	**"*U*" value**	***p* value**	**"*U*" value**	***p* value**	**"*U*" value**	***p* value**
Ctrl vs CPZ	$U = 3.000$	$p = 0.513$	$U = 2.000$	$p = 0.275$	$U = 3.000$	$p = 0.513$	$U = 4.000$	$p = 0.827$
Ctrl vs GNPs	$U = 3.000$	$p = 0.513$	$U = 2.000$	$p = 0.275$	$U = 4.000$	$p = 0.827$	$U = 0.000$	**$p = 0.050$***
Ctrl vs EGF	$U = 1.000$	$p = 0.127$	$U = 2.000$	$p = 0.275$	$U = 3.000$	$p = 0.513$	$U = 4.000$	$p = 0.827$
Ctrl vs EGF-GNPs	$U = 3.000$	$p = 0.513$	$U = 0.000$	**$p = 0.050$***	$U = 0.000$	**$p = 0.050$***	$U = 4.000$	$p = 0.827$
CPZ vs GNPs	$U = 4.000$	$p = 0.827$	$U = 1.000$	$p = 0.127$	$U = 4.000$	$p = 0.827$	$U = 2.000$	$p = 0.275$
CPZ vs EGF	$U = 2.000$	$p = 0.275$	$U = 4.000$	$p = 0.827$	$U = 1.000$	$p = 0.127$	$U = 4.000$	$p = 0.827$
CPZ vs EGF-GNPs	$U = 3.000$	$p = 0.513$	$U = 0.000$	**$p = 0.050$***	$U = 4.000$	$p = 0.827$	$U = 2.000$	$p = 0.275$
GNPs vs EGF	$U = 3.000$	$p = 0.513$	$U = 0.000$	**$p = 0.050$***	$U = 3.000$	$p = 0.513$	$U = 2.000$	$p = 0.275$
GNPs vs EGF-GNPs	$U = 3.000$	$p = 0.513$	$U = 2.000$	$p = 0.275$	$U = 3.000$	$p = 0.513$	$U = 0.000$	**$p = 0.050$***
EGF vs EGF-GNPs	$U = 1.000$	$p = 0.127$	$U = 0.000$	**$p = 0.050$***	$U = 1.000$	$p = 0.127$	$U = 4.000$	$p = 0.827$

Act-B

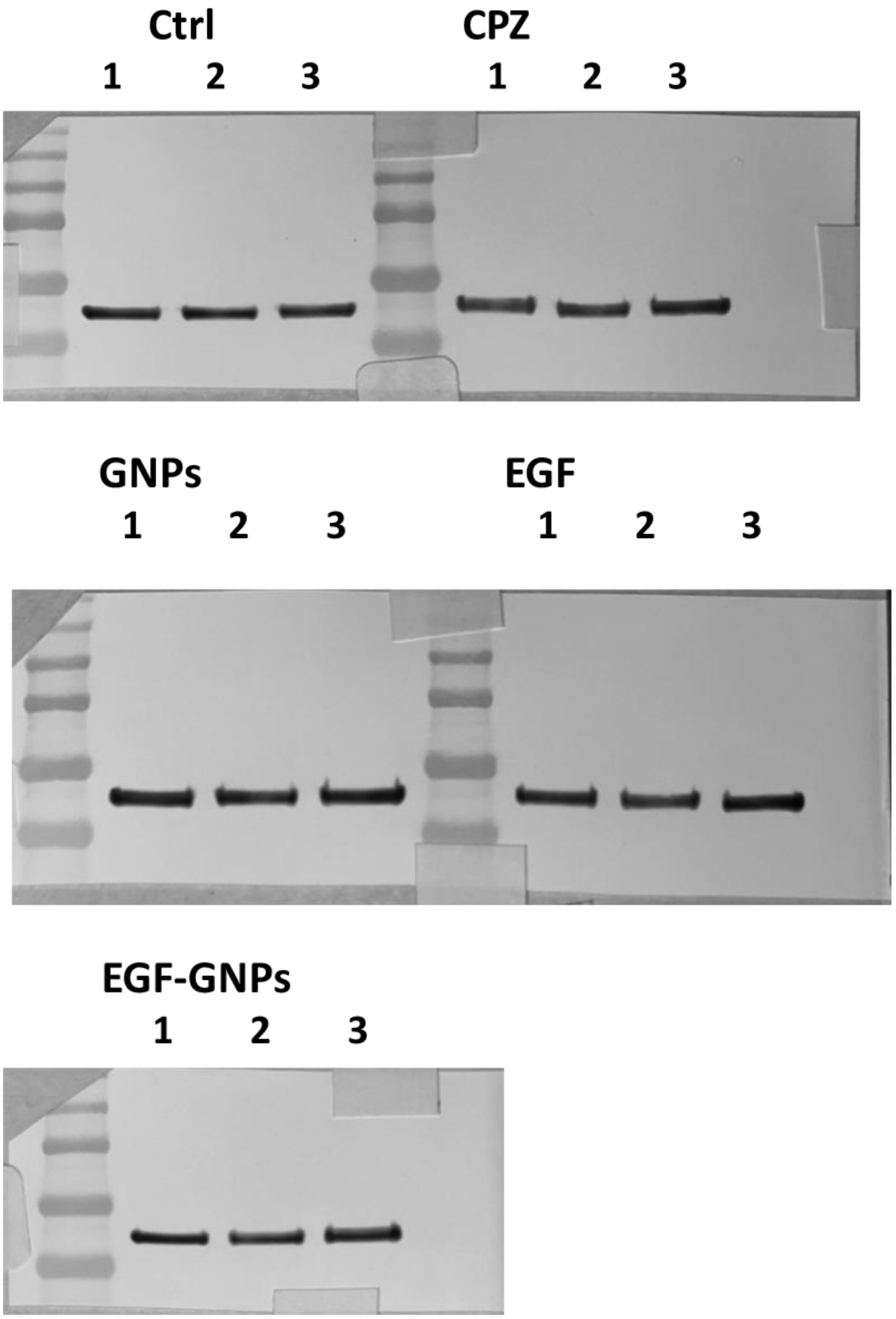

ACT-B: duplicates used as representative images in the main text

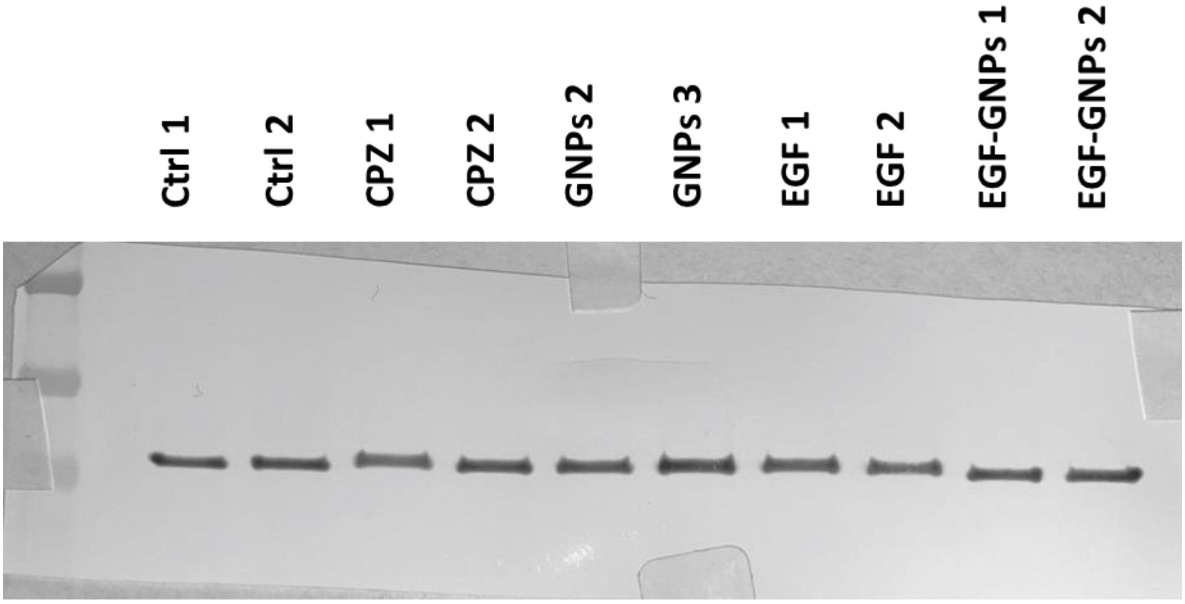
Ctrl 1
Ctrl 2
CPZ 1
CPZ 2
GNPs 2
GNPs 3
EGF 1
EGF 2
EGF-GNPs 1
EGF-GNPs 2

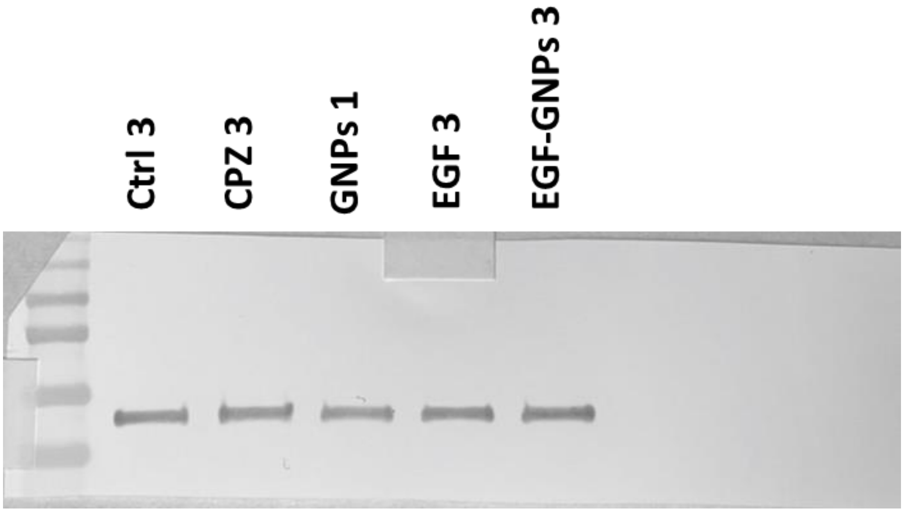
Ctrl 3
CPZ 3
GNPs 1
EGF 3
EGF-GNPs 3

MBP

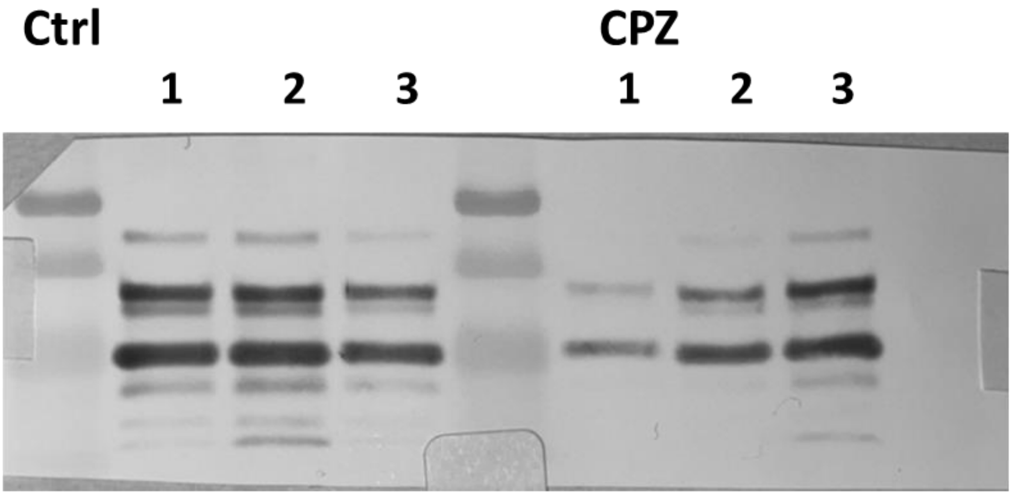
Ctrl
CPZ
1
2
3
1
2
3

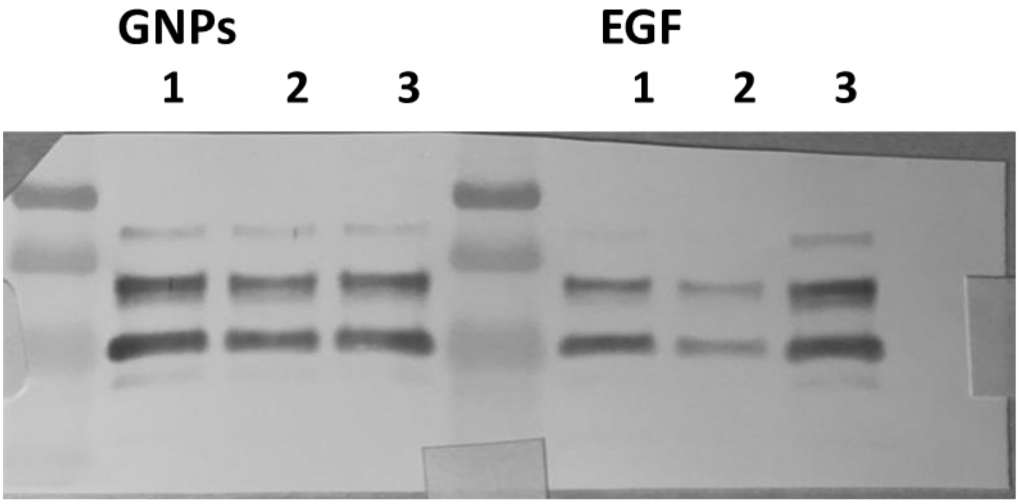

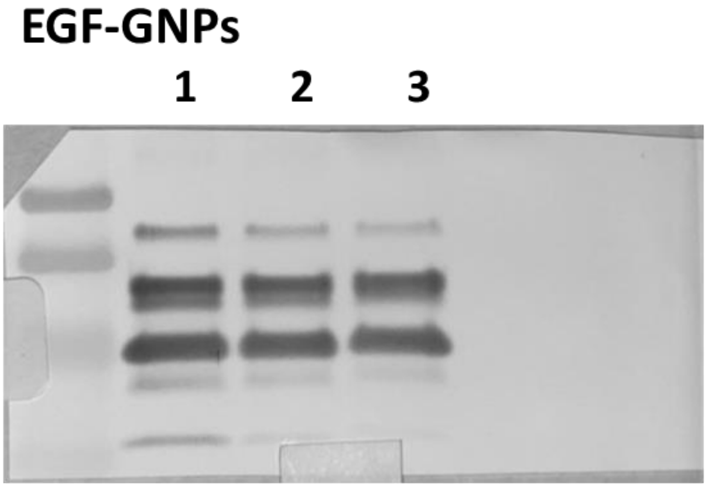

MBP: duplicates used as representative images in the main text

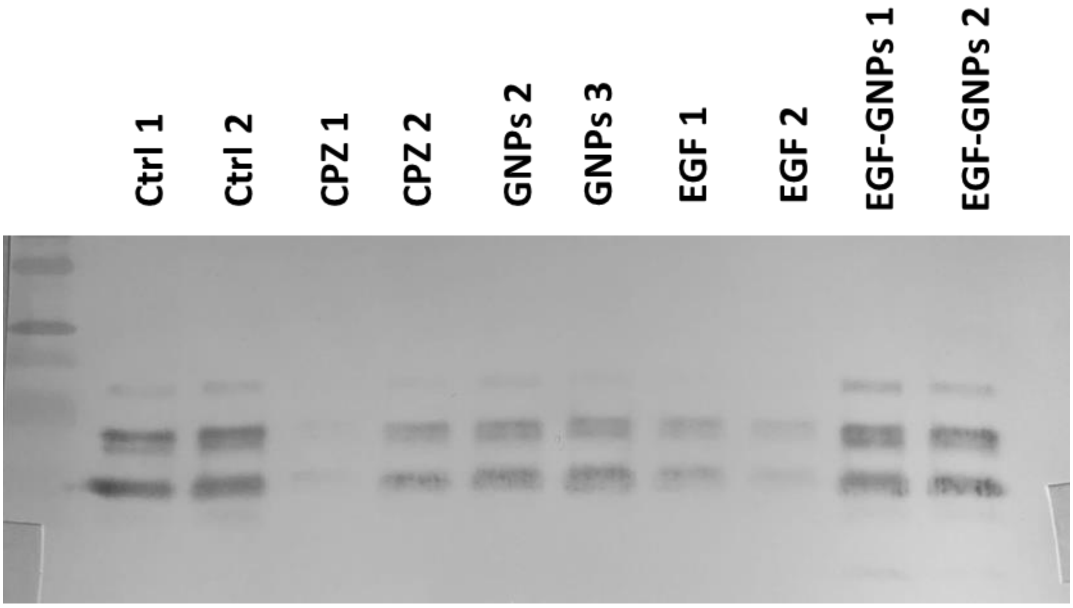

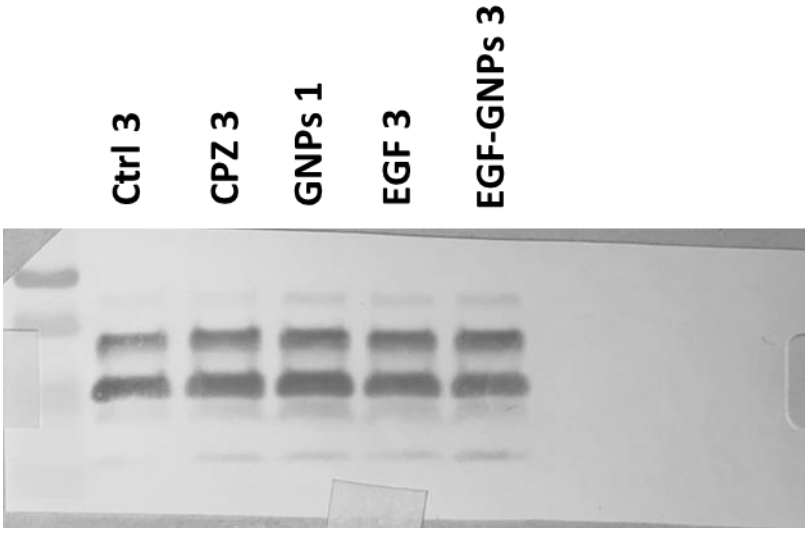
Ctrl 3
CPZ 3
GNPs 1
EGF 3
EGF-GNPs 3

MOG

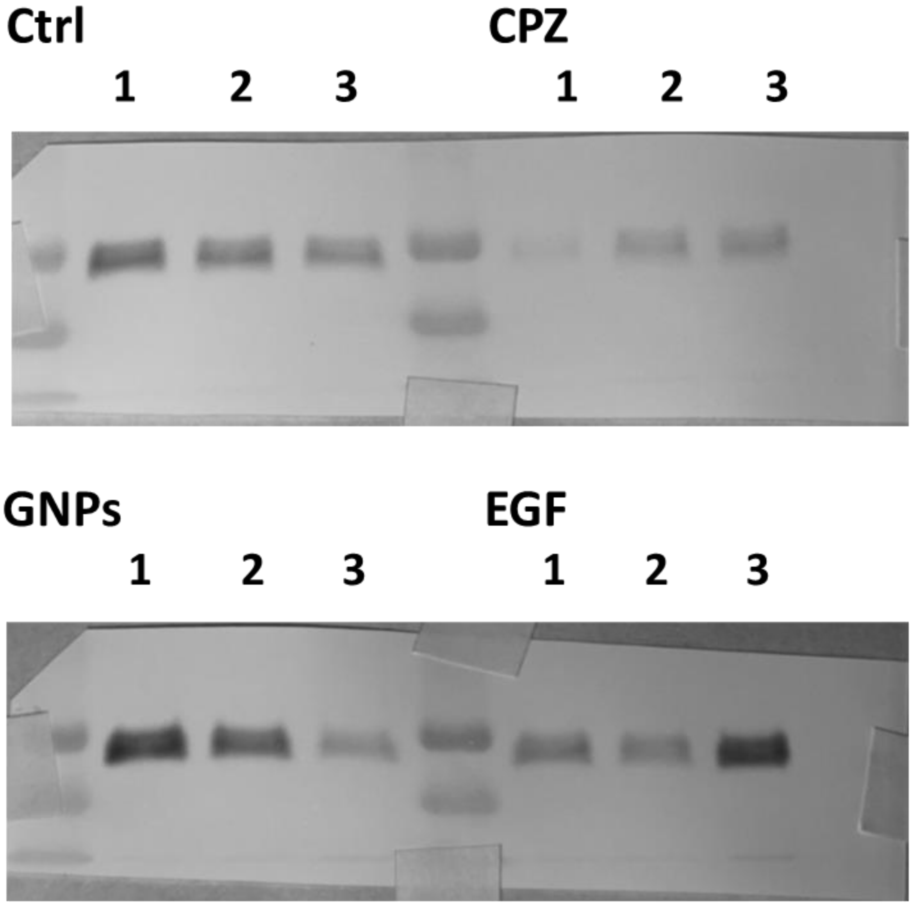
Ctrl
CPZ
1 2 3
1 2 3
GNPs
EGF
1 2 3
1 2 3

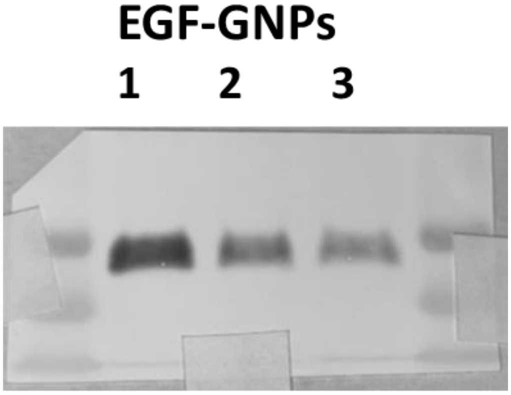

MOG: duplicates used as representative imagess in the main text

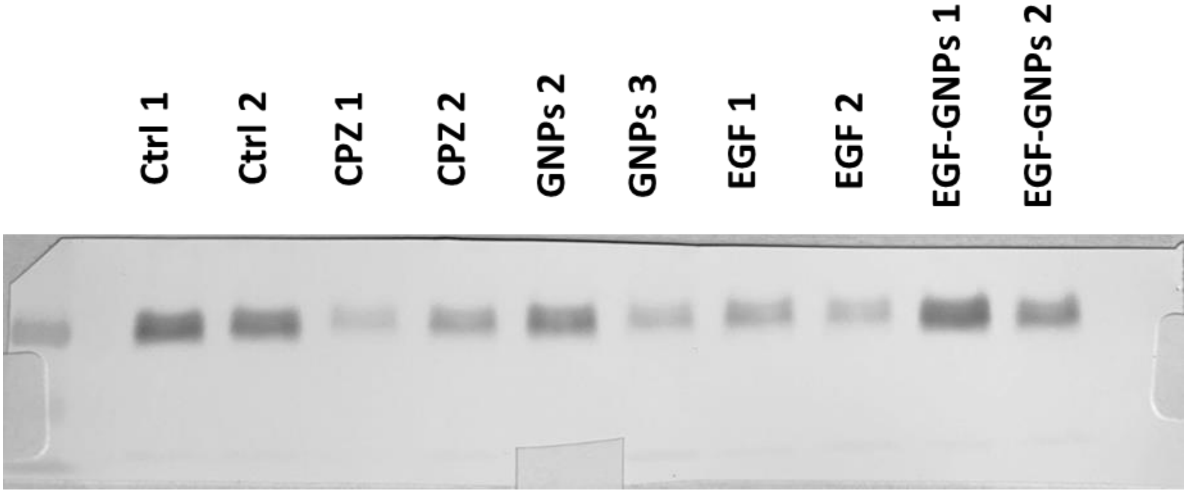

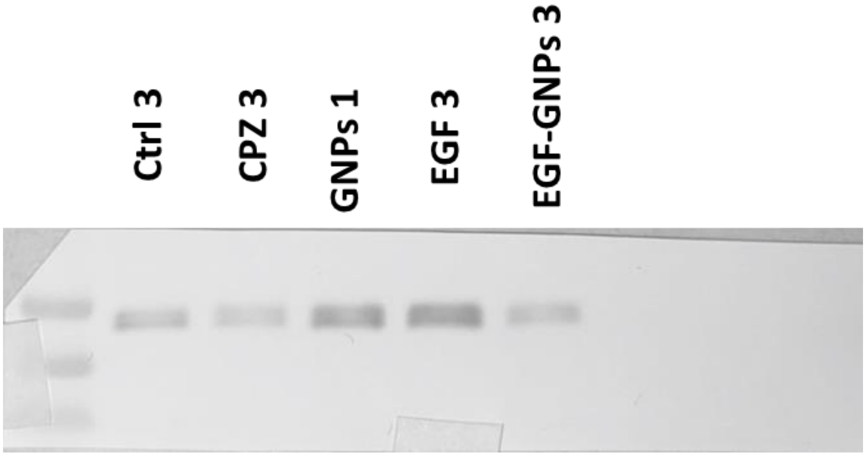

CNPase

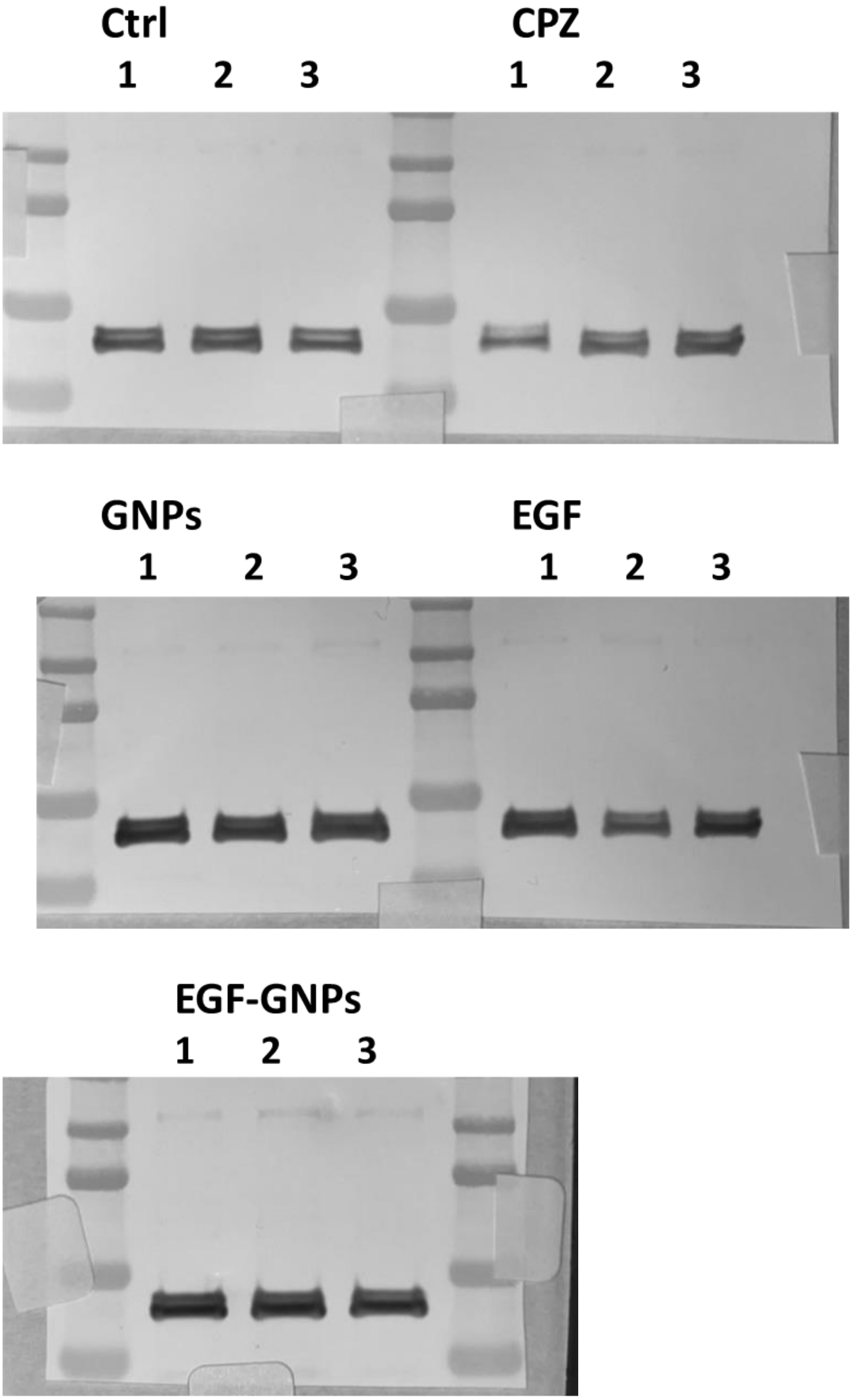

CNPase: duplicates used as representative images in the main text

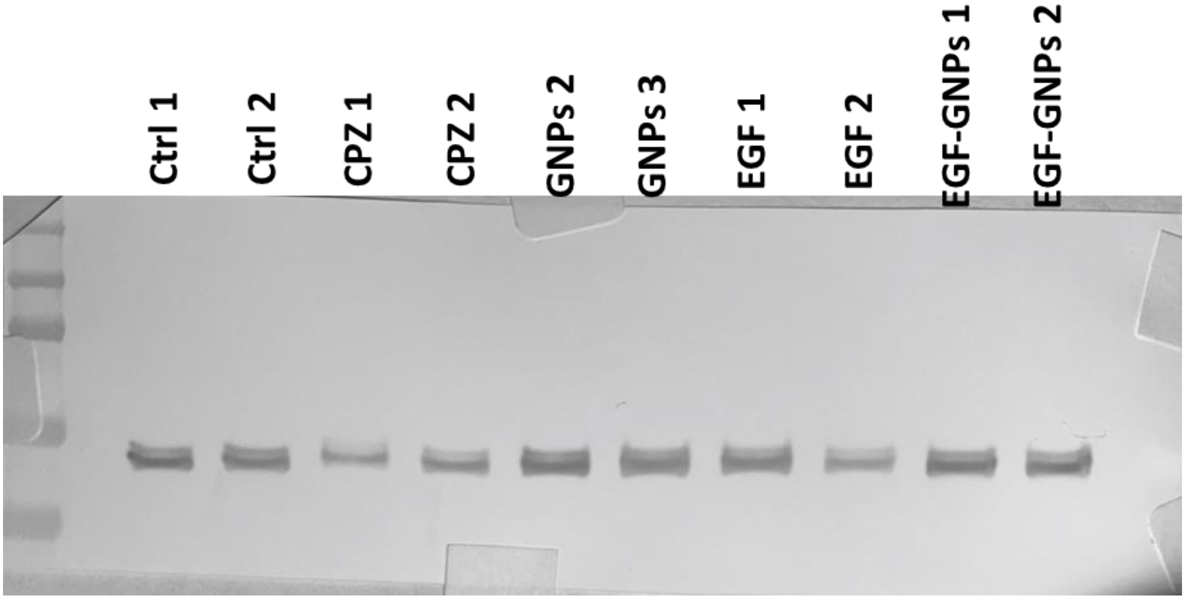
Ctrl 1
Ctrl 2
CPZ 1
CPZ 2
GNPs 2
GNPs 3
EGF 1
EGF 2
EGF-GNPs 1
EGF-GNPs 2

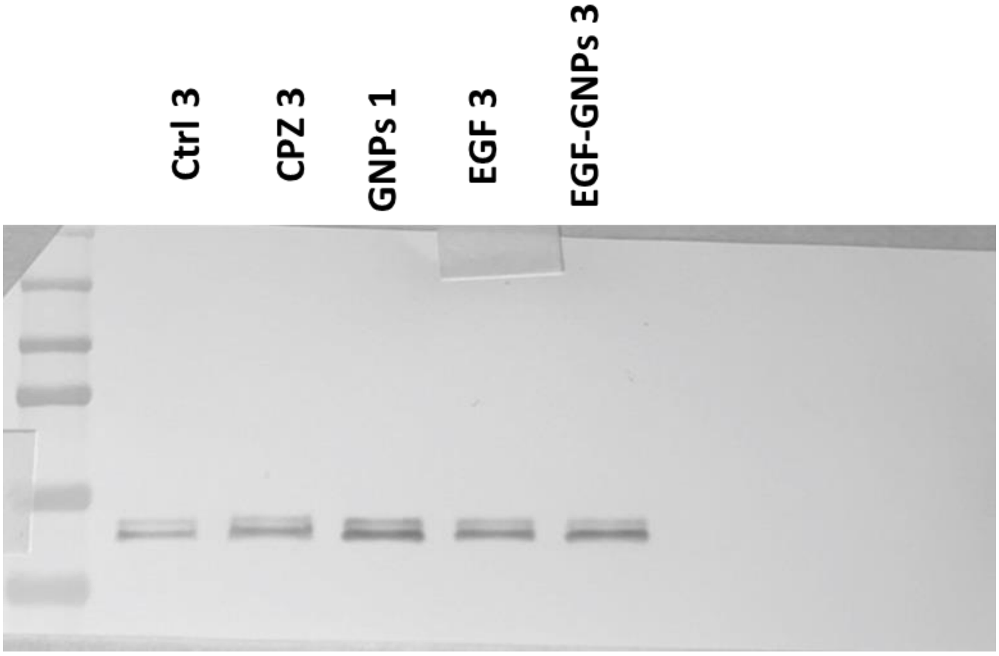
Ctrl 3
CPZ 3
GNPs 1
EGF 3
EGF-GNPs 3

MAG

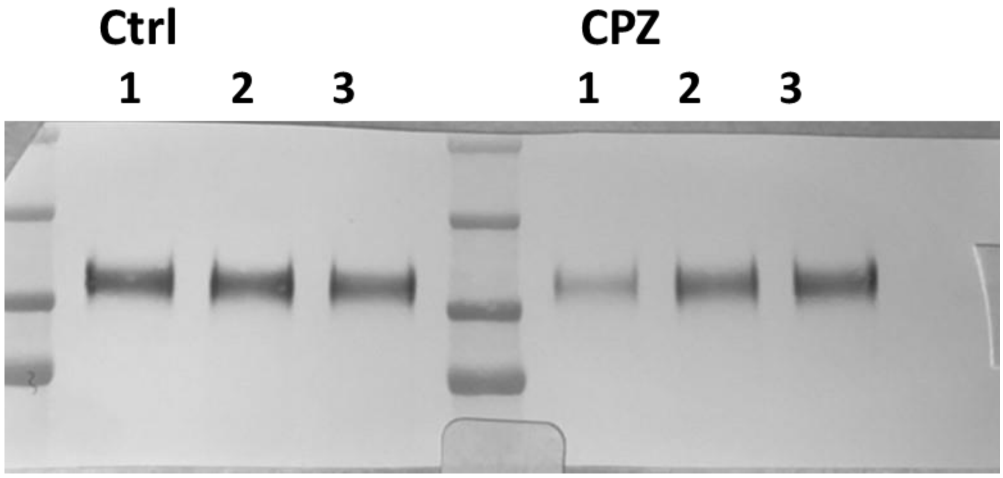

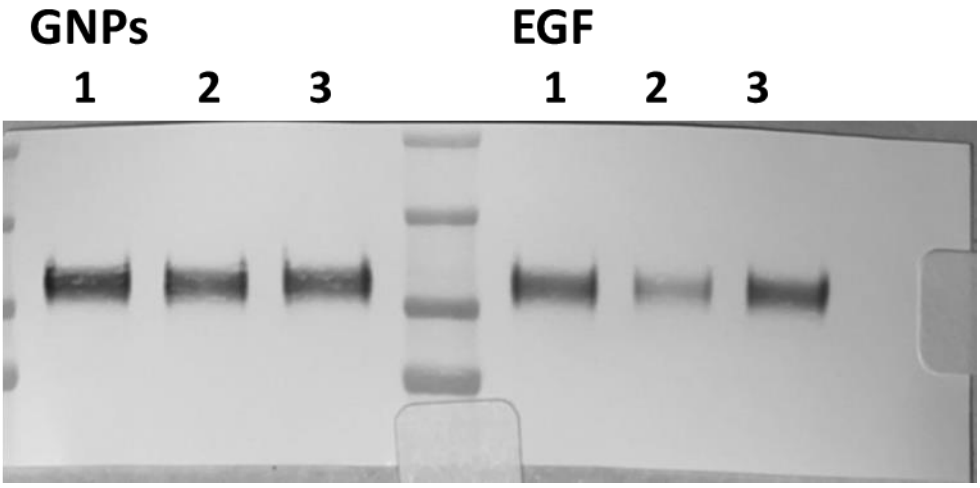

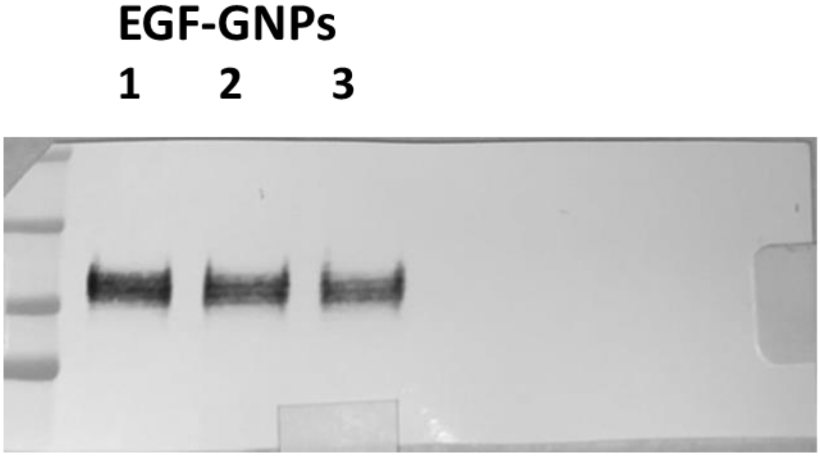

MAG: duplicates used as representative images in the main text

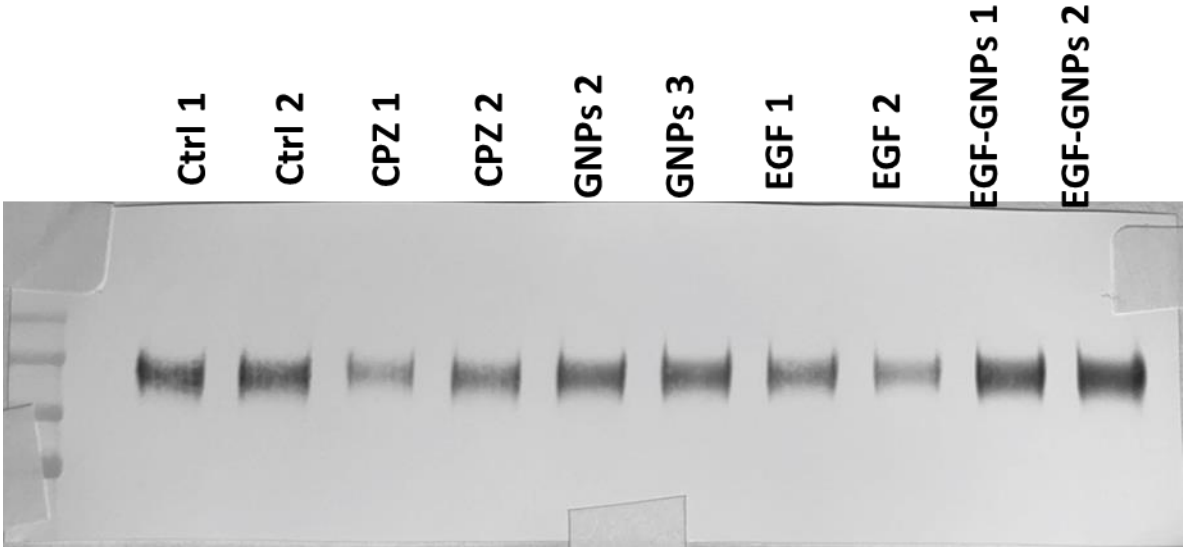

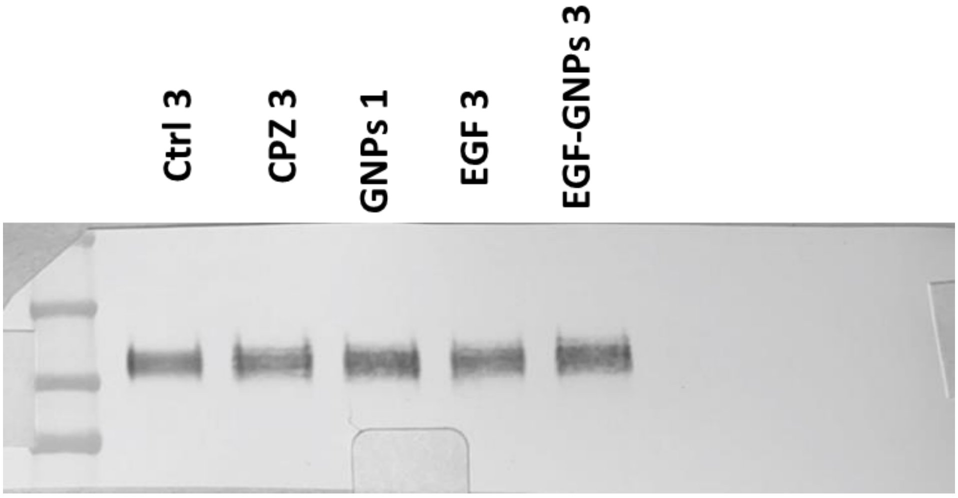

Figure S1. Blots obtained at 2 weeks of recovery. Every subject for each group was assigned with a number from 1 to 3. The duplicates were organized in a different arrangement, we used the numbers to identify each animal in every blot.

Act-B

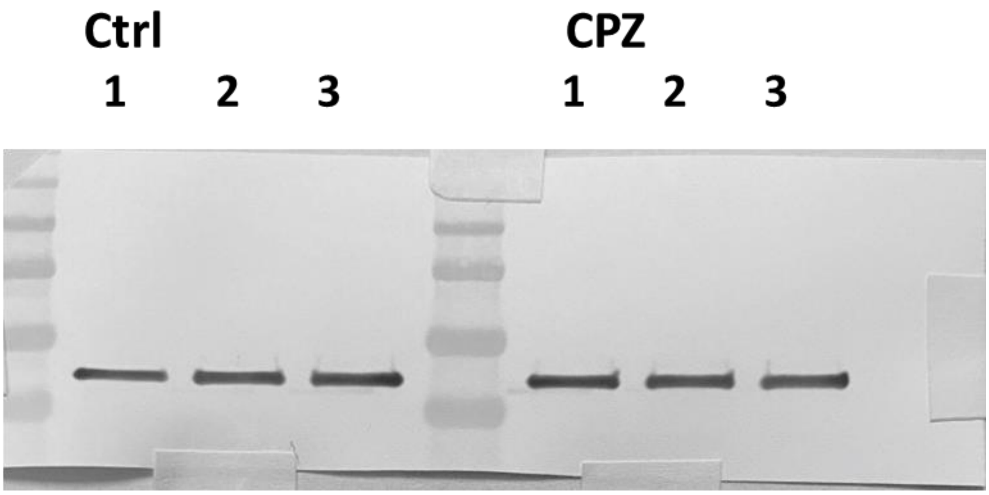

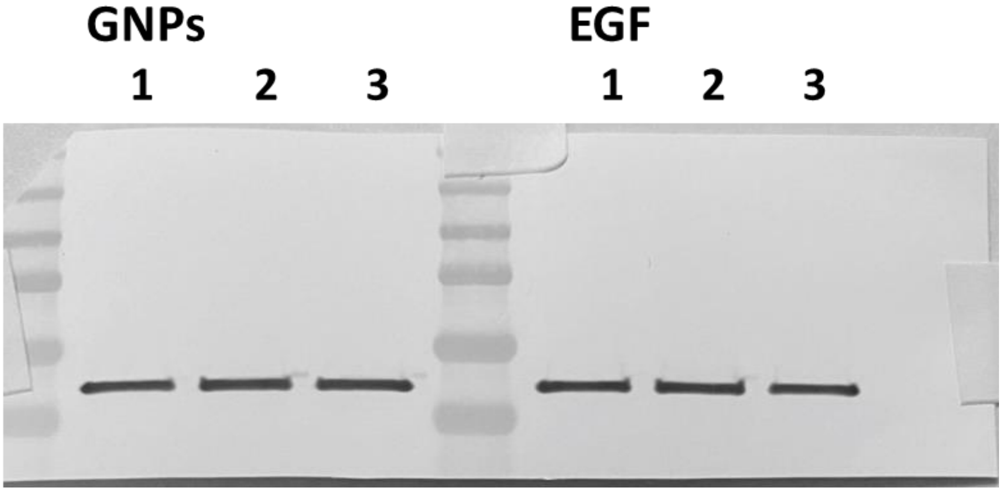

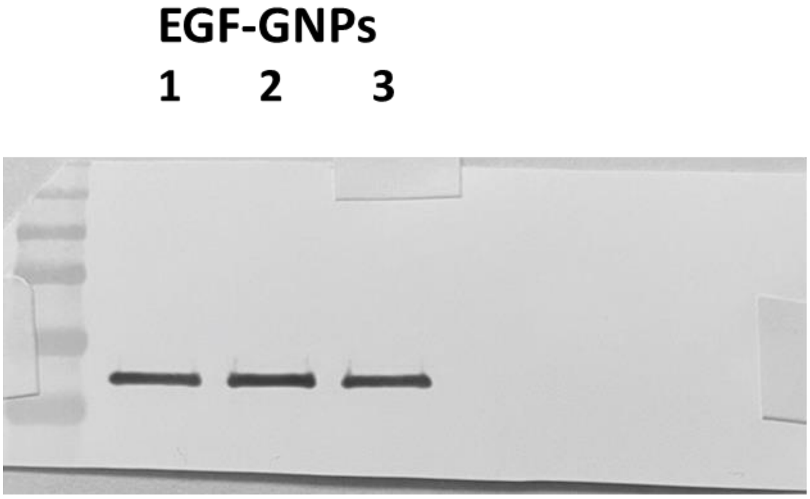

Act-B: duplicates used as representative images in the main text.

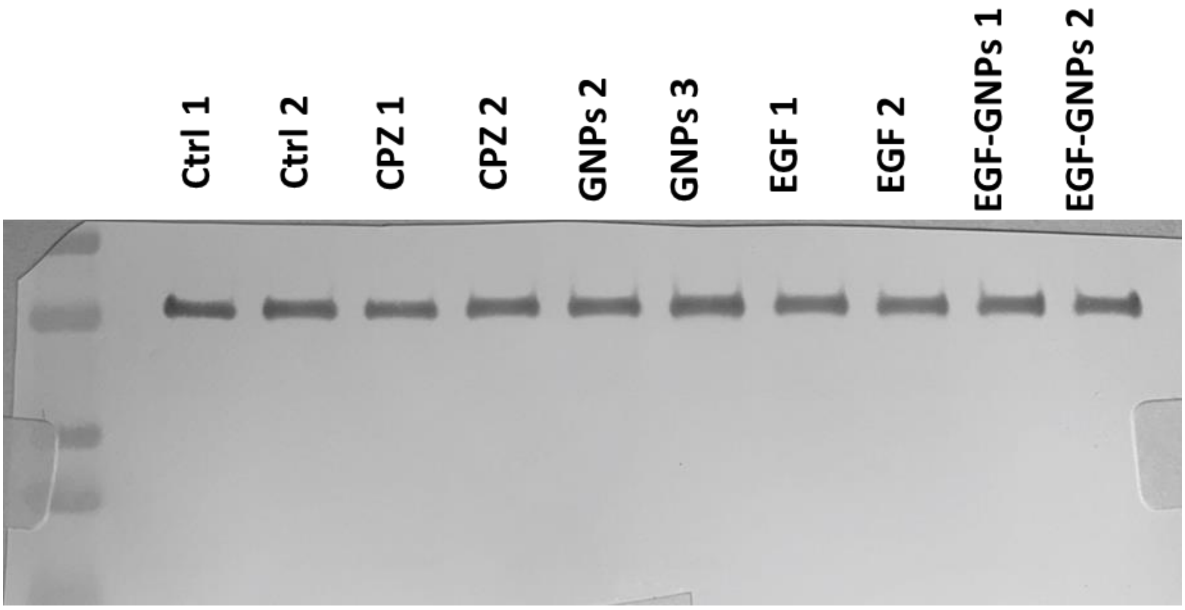

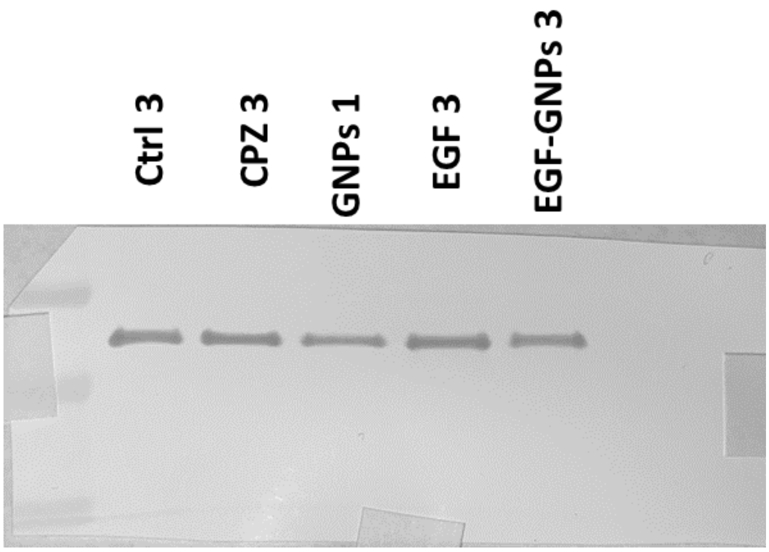
Ctrl 3
CPZ 3
GNPs 1
EGF 3
EGF-GNPs 3

MBP

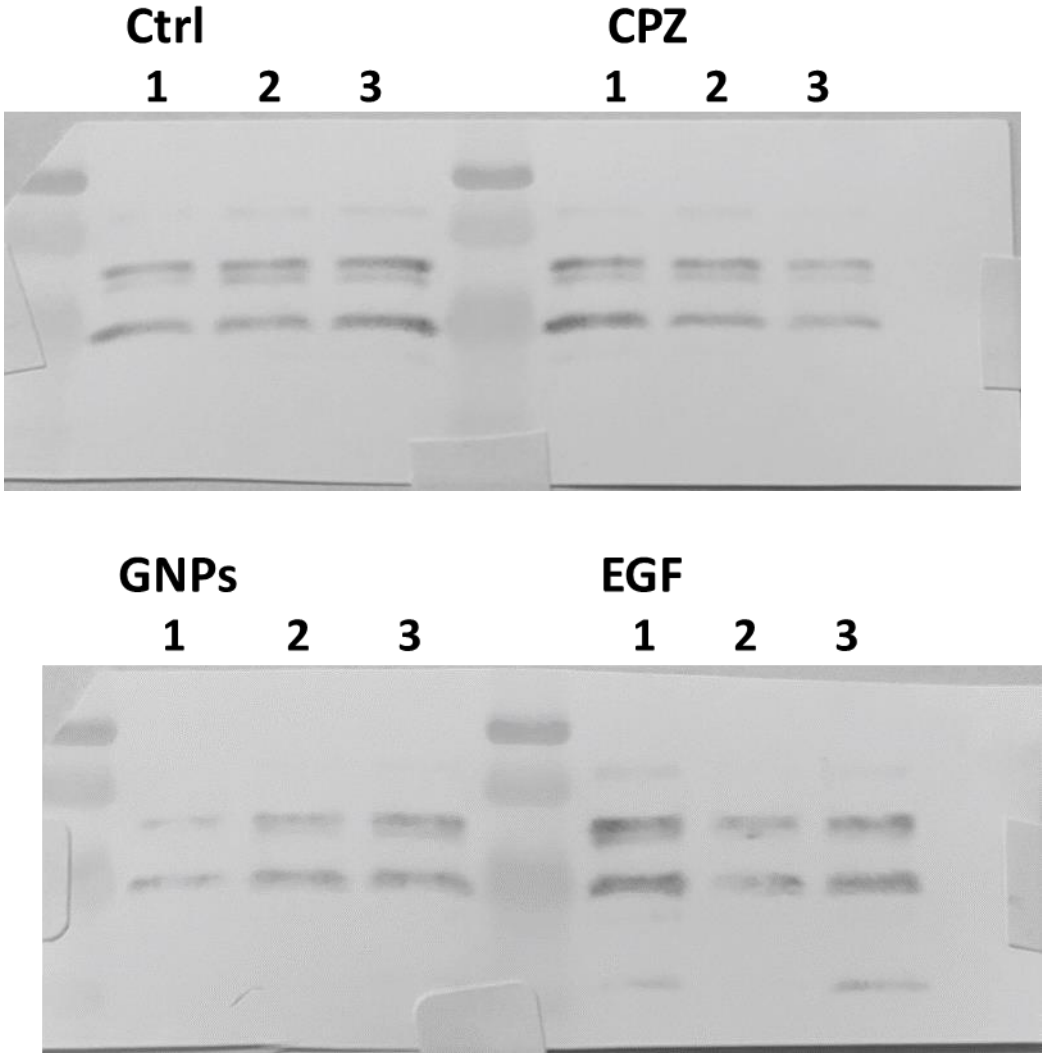
Ctrl
1 2 3
CPZ
1 2 3
GNPs
1 2 3
EGF
1 2 3

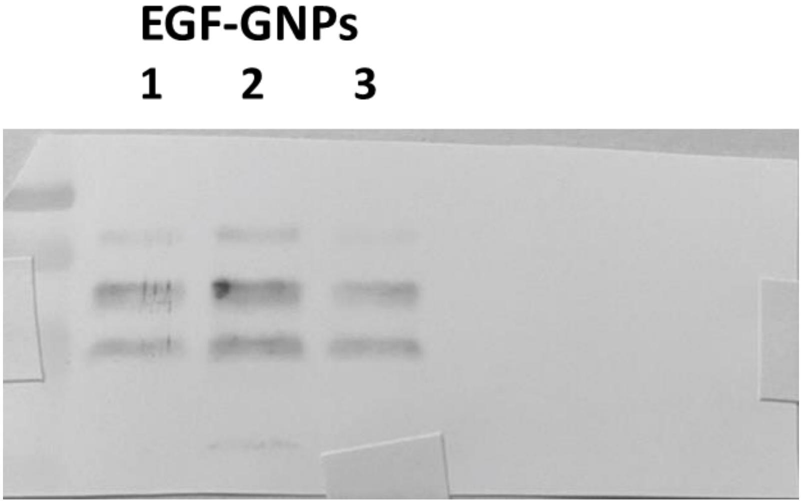

MBP: duplicates used as representative images in the main text

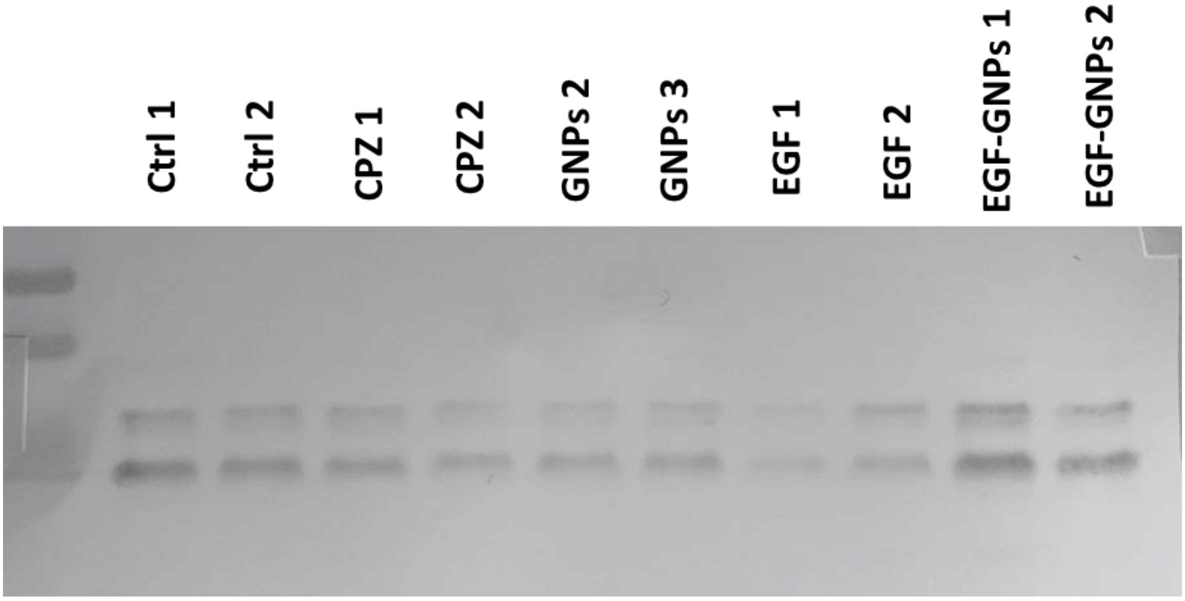

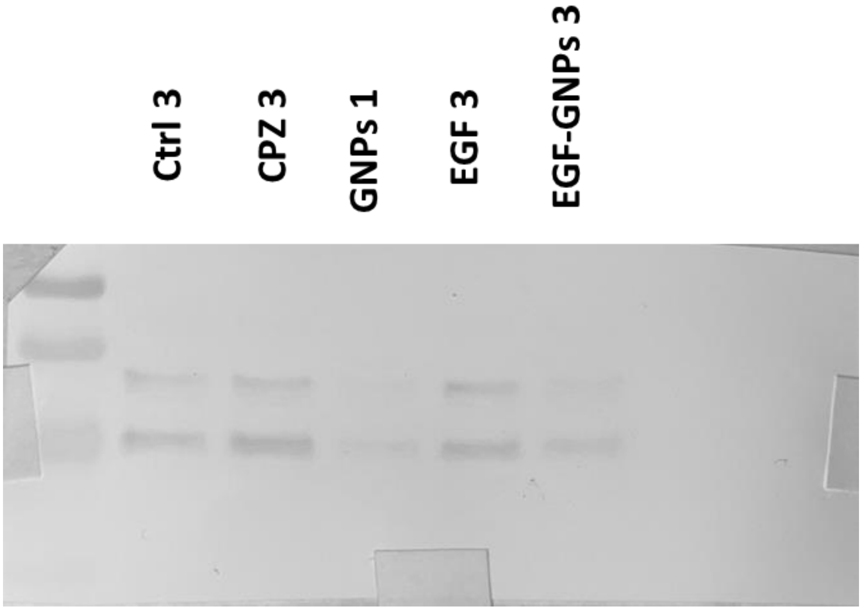
Ctrl 3
CPZ 3
GNPs 1
EGF 3
EGF-GNPs 3

MOG

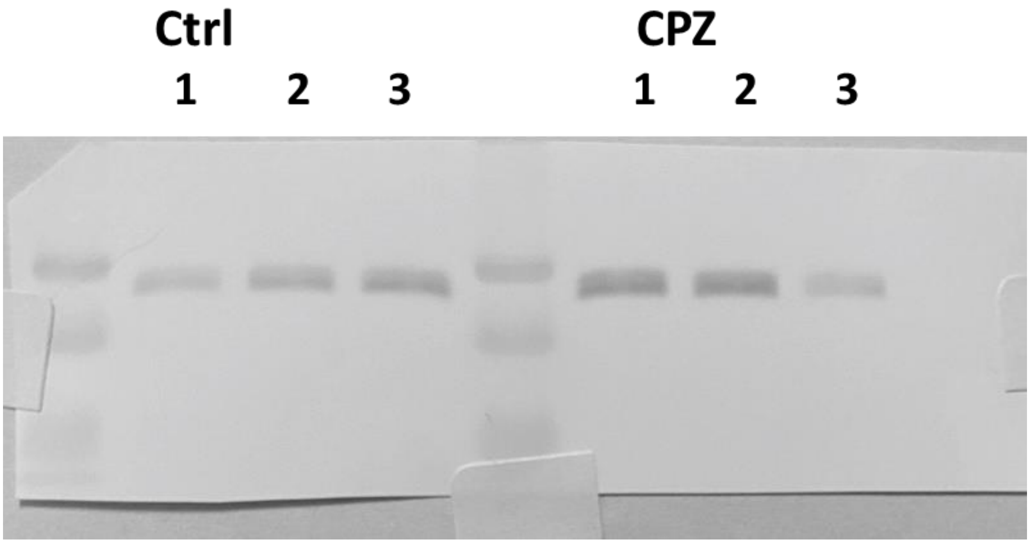
Ctrl
1 2 3
CPZ
1 2 3

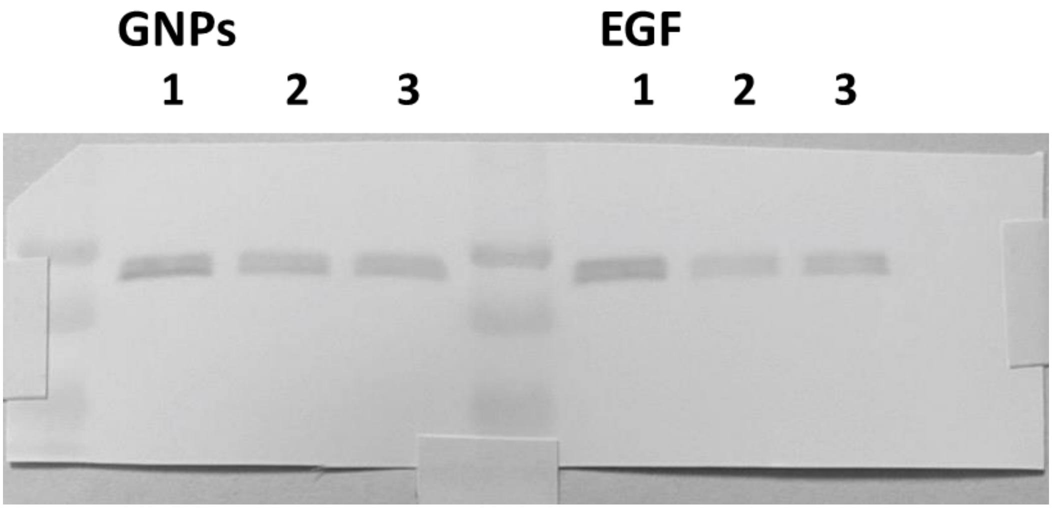
GNPs
1 2 3
EGF
1 2 3

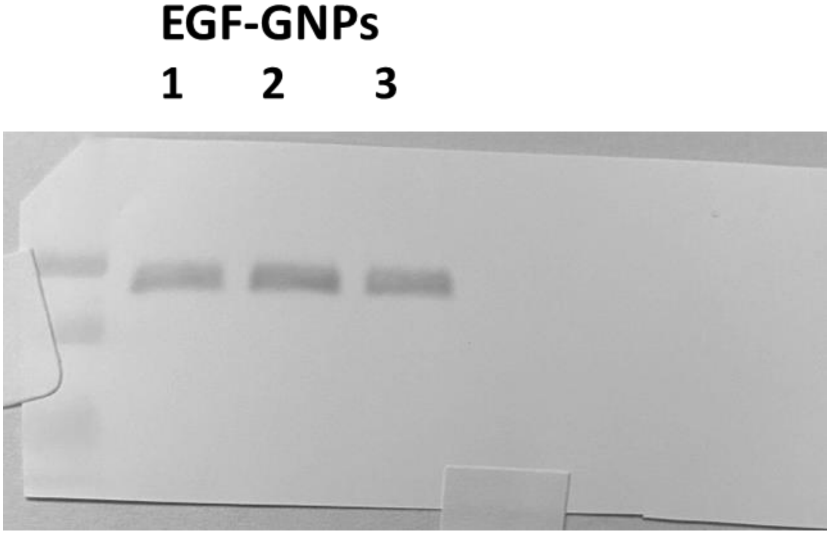

MOG: duplicates used as representative images in the main text

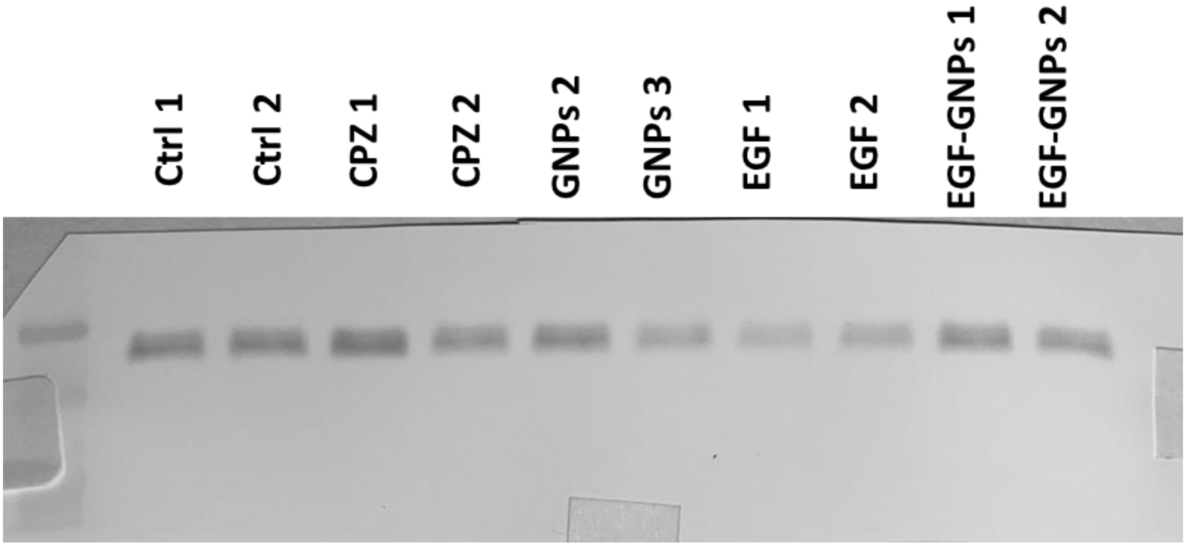

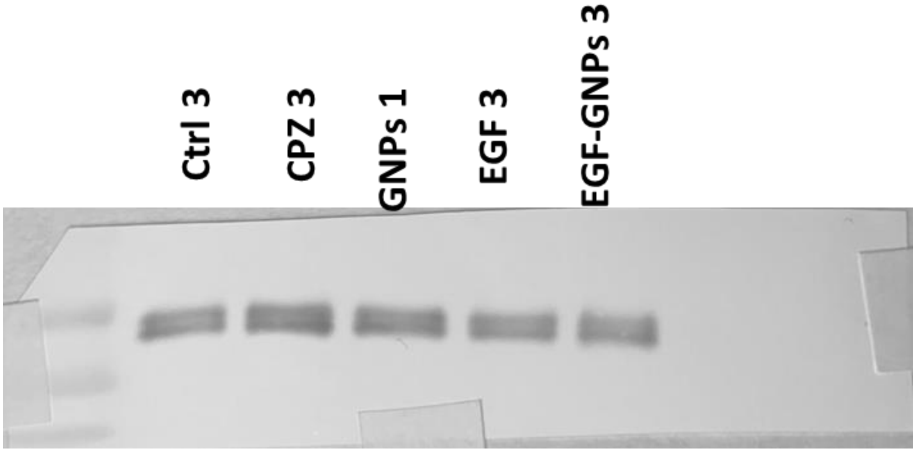

CNPase

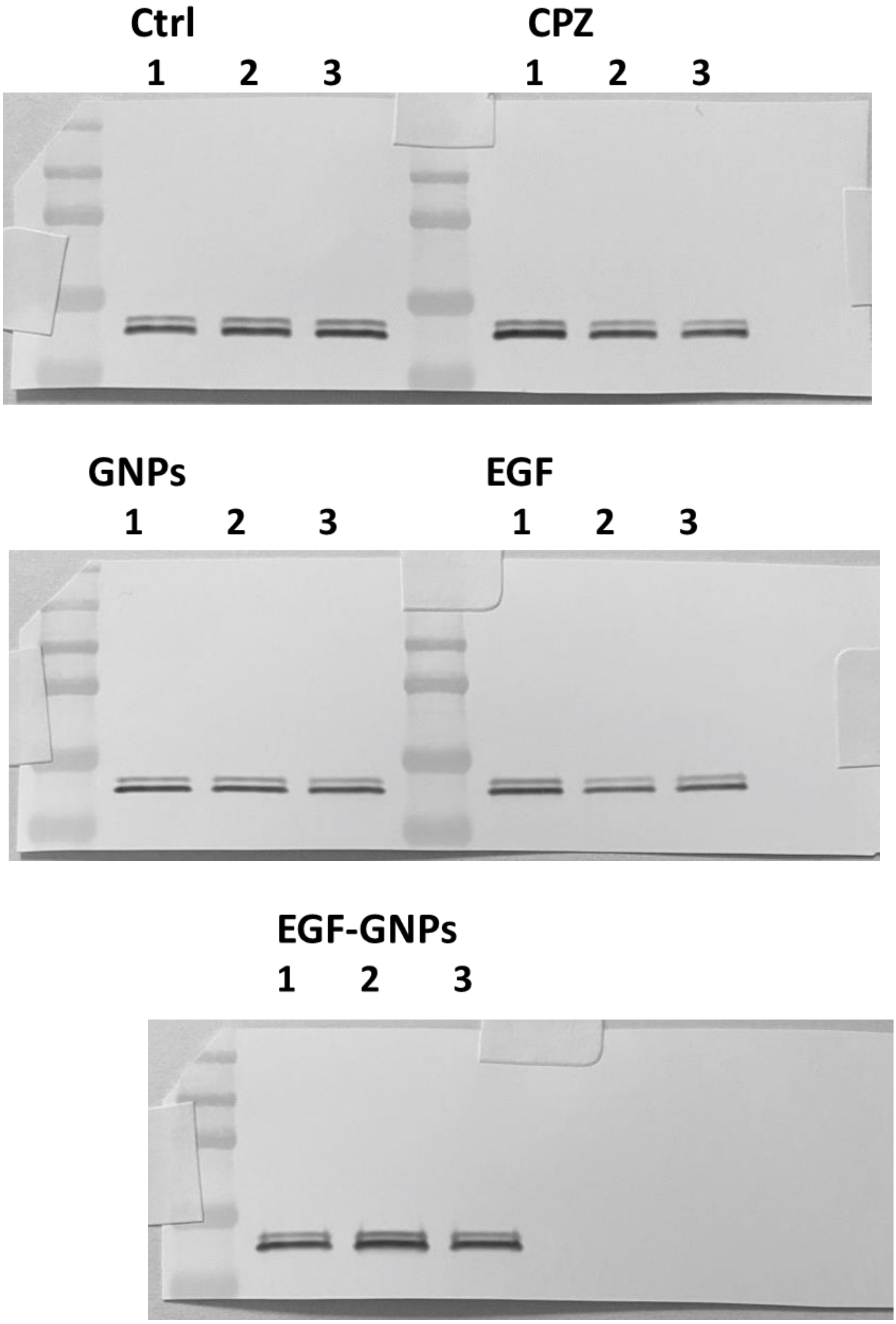

CNPase: duplicates used as representative images in the main text

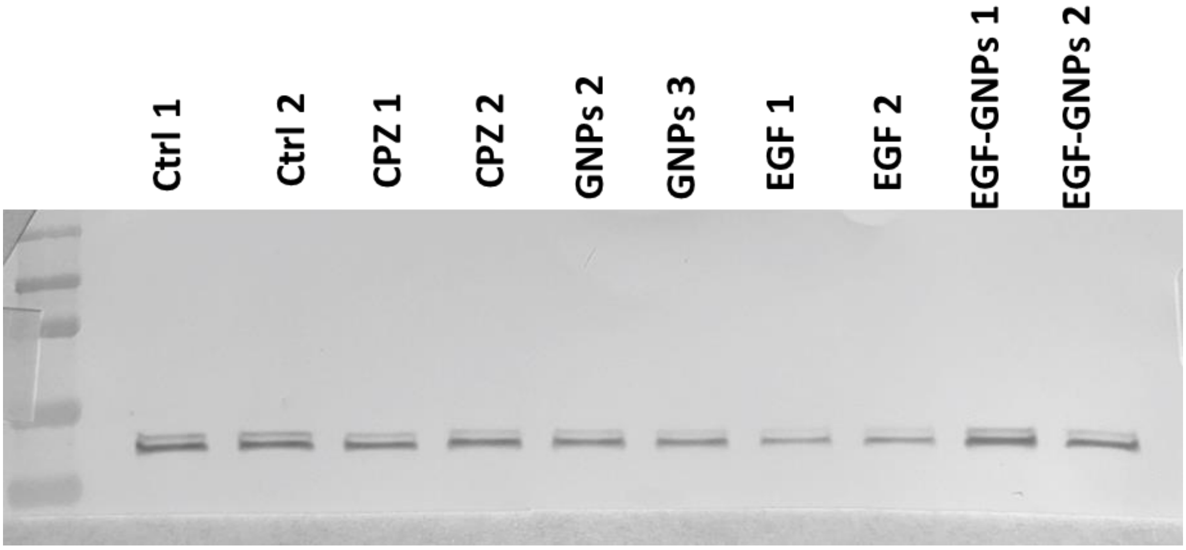
Ctrl 1
Ctrl 2
CPZ 1
CPZ 2
GNPs 2
GNPs 3
EGF 1
EGF 2
EGF-GNPs 1
EGF-GNPs 2

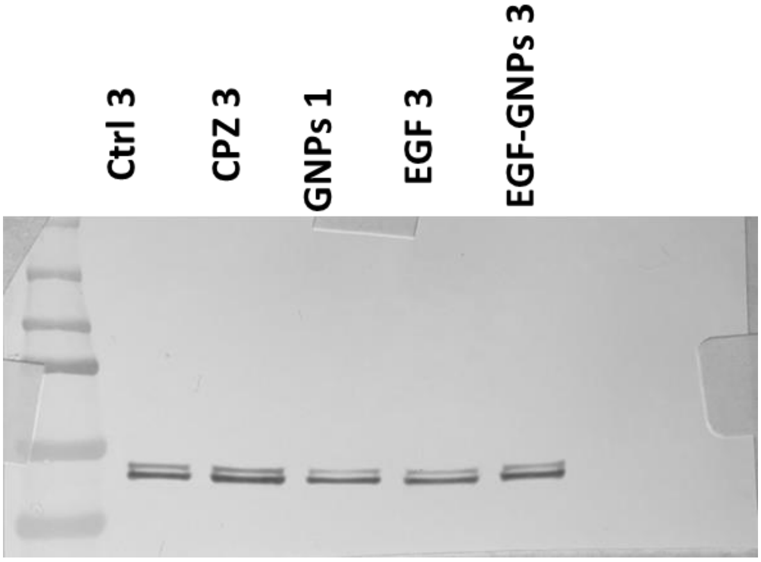
Ctrl 3
CPZ 3
GNPs 1
EGF 3
EGF-GNPs 3

MAG

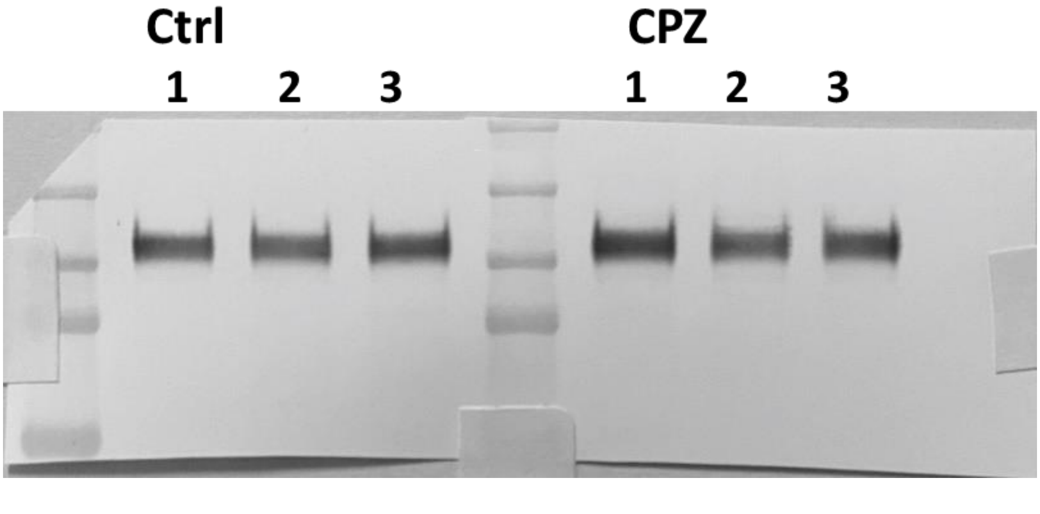
Ctrl
1
2
3
CPZ
1
2
3

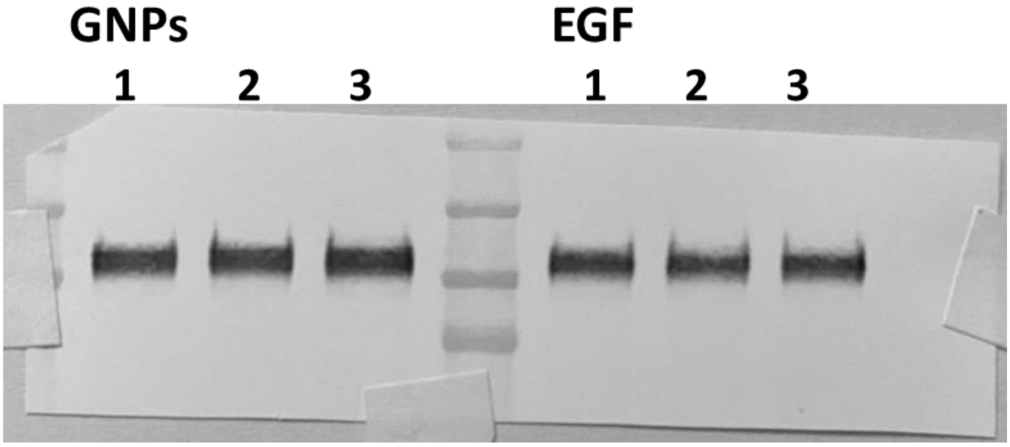

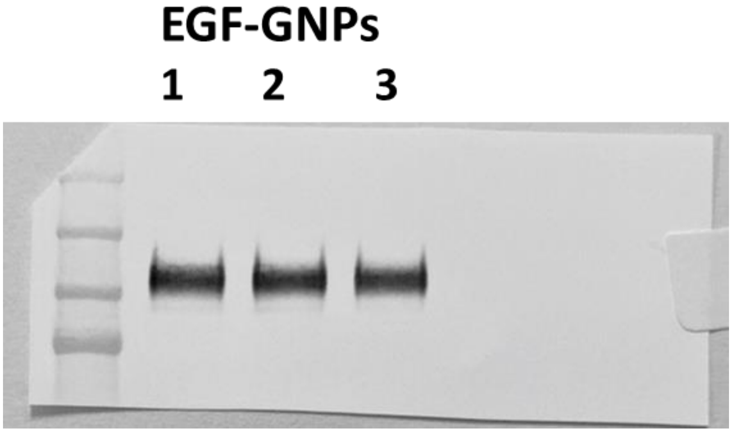

MAG: duplicates used as representative images in the main text

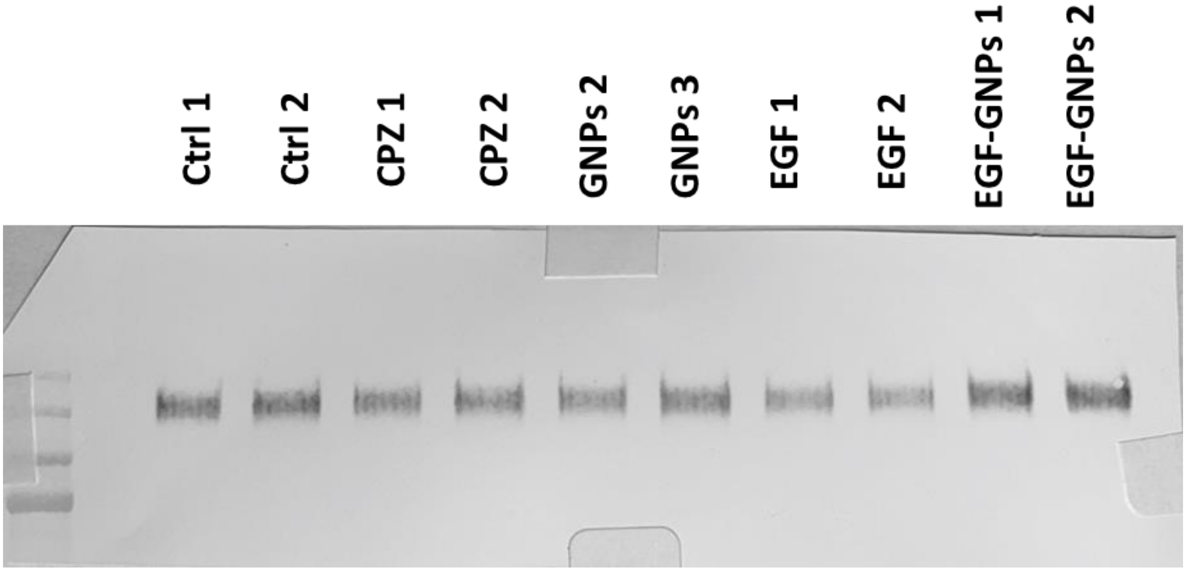

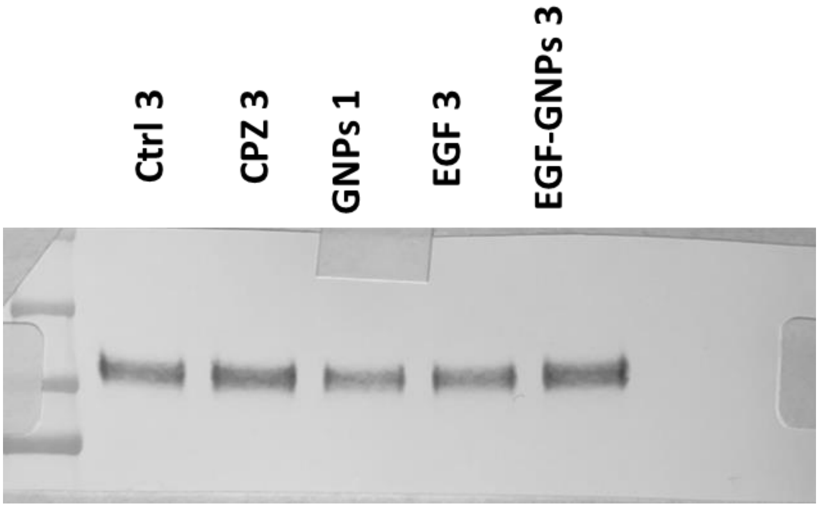

Figure S2. Blots obtained at 3 weeks of recovery.
